# Supplementary figures and images for: TTC13 expression and STAT3 activation may form a positive feedback loop to promote ccRCC progression
Source: PeerJ. 2023 Oct 30;11:e16316. doi: 10.7717/peerj.16316 (PMC10621595; doi:10.7717/peerj.16316)

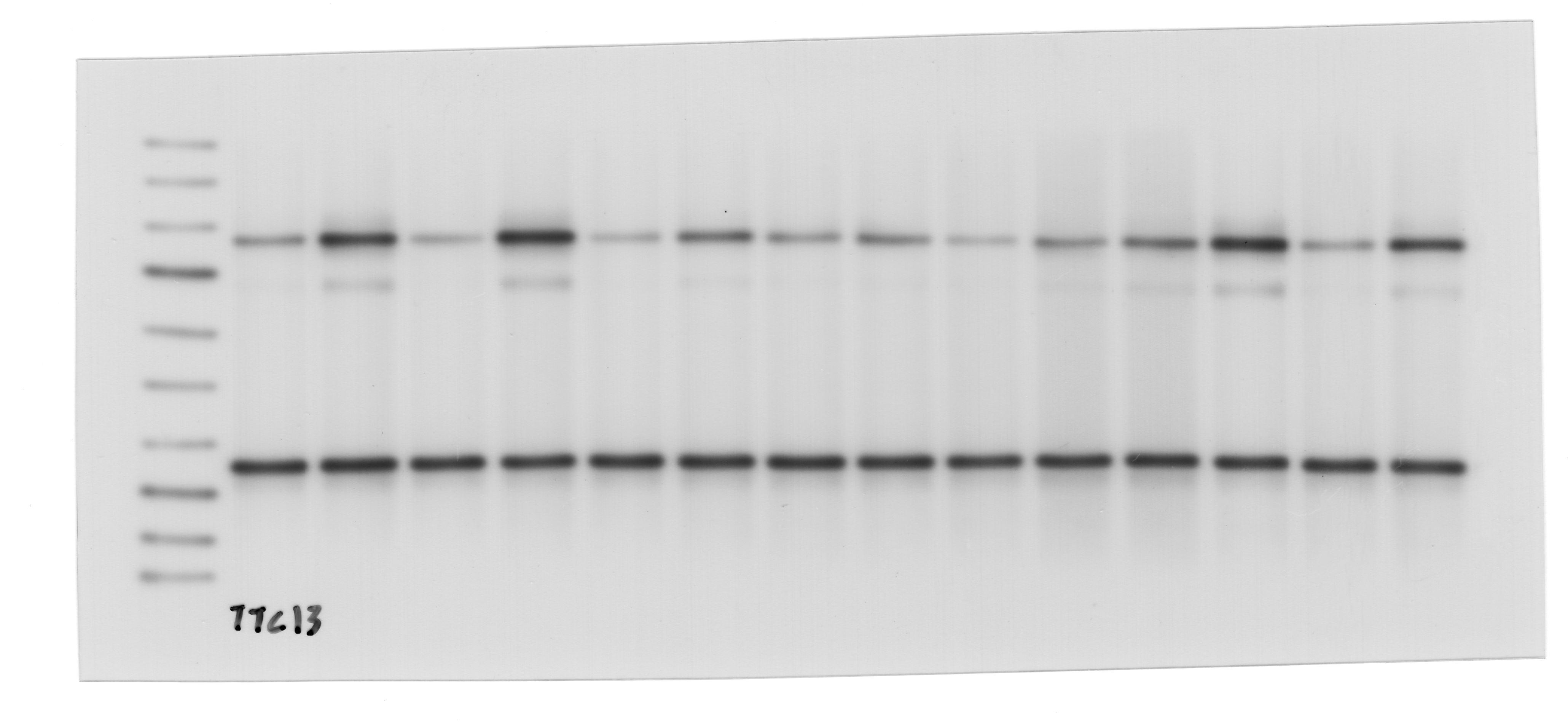

Supplement: Supplemental Information 1 — The original image of WB. [file peerj-11-16316-s001.zip › Original Images for Blots Gels/Figure1/F1-E.tif]

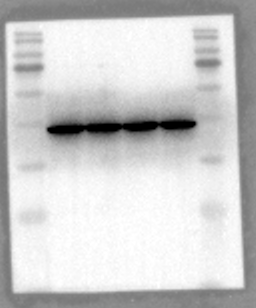

Supplement: Supplemental Information 1 — The original image of WB. [file peerj-11-16316-s001.zip › Original Images for Blots Gels/Figure1/F1-G-ACTIN.Tif]

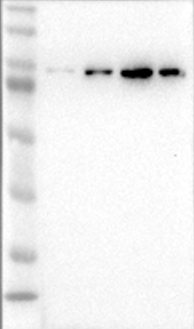

Supplement: Supplemental Information 1 — The original image of WB. [file peerj-11-16316-s001.zip › Original Images for Blots Gels/Figure1/Fig1G-TTC13.Tif]

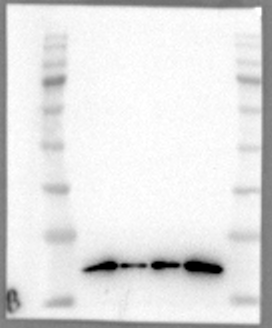

Supplement: Supplemental Information 1 — The original image of WB. [file peerj-11-16316-s001.zip › Original Images for Blots Gels/Figure2/F2-C/786-0/F2-C-BAX.Tif]

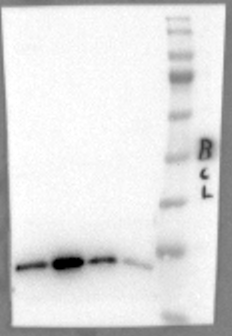

Supplement: Supplemental Information 1 — The original image of WB. [file peerj-11-16316-s001.zip › Original Images for Blots Gels/Figure2/F2-C/786-0/F2-C-BCL-2.Tif]

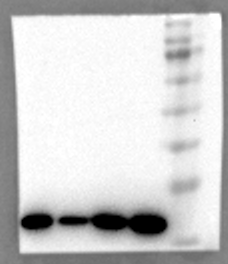

Supplement: Supplemental Information 1 — The original image of WB. [file peerj-11-16316-s001.zip › Original Images for Blots Gels/Figure2/F2-C/786-0/F2-C-CASPASE3.Tif]

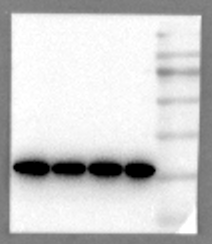

Supplement: Supplemental Information 1 — The original image of WB. [file peerj-11-16316-s001.zip › Original Images for Blots Gels/Figure2/F2-C/786-0/F2-C-GAPDH.Tif]

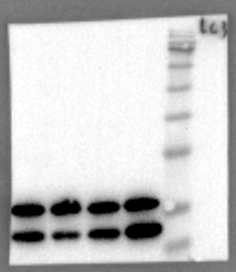

Supplement: Supplemental Information 1 — The original image of WB. [file peerj-11-16316-s001.zip › Original Images for Blots Gels/Figure2/F2-C/786-0/F2-C-LC3.Tif]

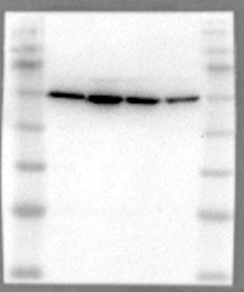

Supplement: Supplemental Information 1 — The original image of WB. [file peerj-11-16316-s001.zip › Original Images for Blots Gels/Figure2/F2-C/786-0/F2-C-P62.Tif]

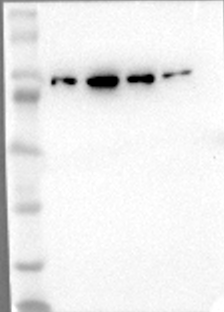

Supplement: Supplemental Information 1 — The original image of WB. [file peerj-11-16316-s001.zip › Original Images for Blots Gels/Figure2/F2-C/786-0/Fig2C-786-0-TTC13.Tif]

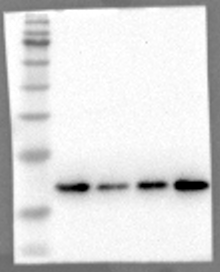

Supplement: Supplemental Information 1 — The original image of WB. [file peerj-11-16316-s001.zip › Original Images for Blots Gels/Figure2/F2-C/caki-1/F2-C-BAX.Tif]

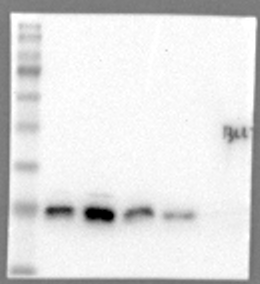

Supplement: Supplemental Information 1 — The original image of WB. [file peerj-11-16316-s001.zip › Original Images for Blots Gels/Figure2/F2-C/caki-1/F2-C-BCL-2.Tif]

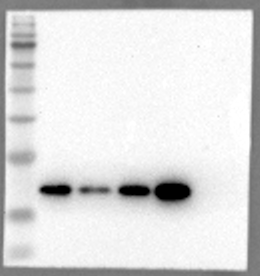

Supplement: Supplemental Information 1 — The original image of WB. [file peerj-11-16316-s001.zip › Original Images for Blots Gels/Figure2/F2-C/caki-1/F2-C-CASPASE3.Tif]

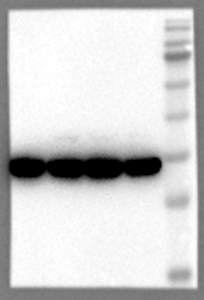

Supplement: Supplemental Information 1 — The original image of WB. [file peerj-11-16316-s001.zip › Original Images for Blots Gels/Figure2/F2-C/caki-1/F2-C-GAPDH.Tif]

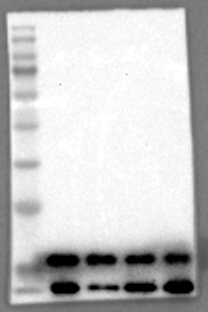

Supplement: Supplemental Information 1 — The original image of WB. [file peerj-11-16316-s001.zip › Original Images for Blots Gels/Figure2/F2-C/caki-1/F2-C-LC3.Tif]

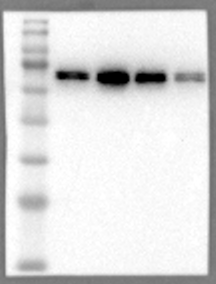

Supplement: Supplemental Information 1 — The original image of WB. [file peerj-11-16316-s001.zip › Original Images for Blots Gels/Figure2/F2-C/caki-1/F2-C-P62.Tif]

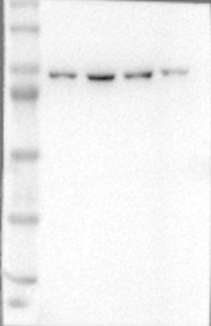

Supplement: Supplemental Information 1 — The original image of WB. [file peerj-11-16316-s001.zip › Original Images for Blots Gels/Figure2/F2-C/caki-1/Fig2C-Caki-1-TTC13.Tif]

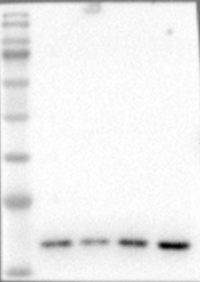

Supplement: Supplemental Information 1 — The original image of WB. [file peerj-11-16316-s001.zip › Original Images for Blots Gels/Figure2/F2-C/HK-2/Fig2C-HK-2 BAX.Tif]

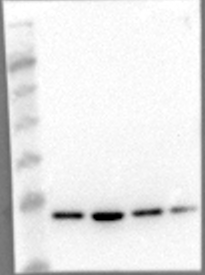

Supplement: Supplemental Information 1 — The original image of WB. [file peerj-11-16316-s001.zip › Original Images for Blots Gels/Figure2/F2-C/HK-2/Fig2C-HK-2 BCL-2.Tif]

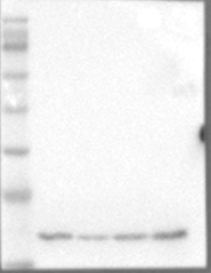

Supplement: Supplemental Information 1 — The original image of WB. [file peerj-11-16316-s001.zip › Original Images for Blots Gels/Figure2/F2-C/HK-2/Fig2C-HK-2 caspase3.Tif]

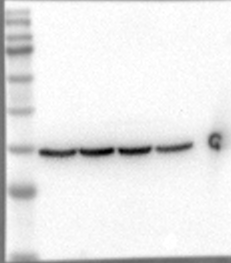

Supplement: Supplemental Information 1 — The original image of WB. [file peerj-11-16316-s001.zip › Original Images for Blots Gels/Figure2/F2-C/HK-2/Fig2C-HK-2 GAPDH.Tif]

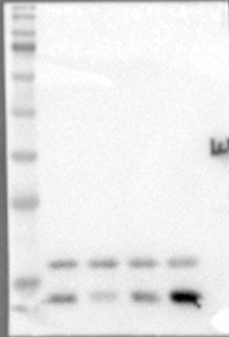

Supplement: Supplemental Information 1 — The original image of WB. [file peerj-11-16316-s001.zip › Original Images for Blots Gels/Figure2/F2-C/HK-2/Fig2C-HK-2 LC3ó≥ó±.Tif]

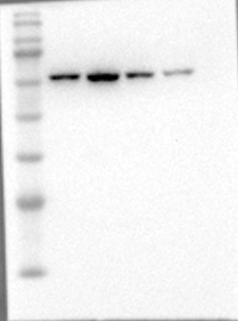

Supplement: Supplemental Information 1 — The original image of WB. [file peerj-11-16316-s001.zip › Original Images for Blots Gels/Figure2/F2-C/HK-2/Fig2C-HK-2 P62.Tif]

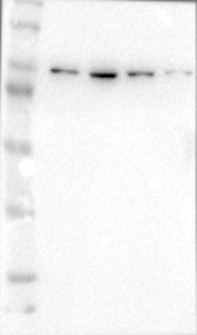

Supplement: Supplemental Information 1 — The original image of WB. [file peerj-11-16316-s001.zip › Original Images for Blots Gels/Figure2/F2-C/HK-2/Fig2C-HK-2-TTC13.Tif]

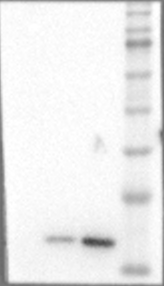

Supplement: Supplemental Information 1 — The original image of WB. [file peerj-11-16316-s001.zip › Original Images for Blots Gels/Figure3/Fig3F-caspase3.Tif]

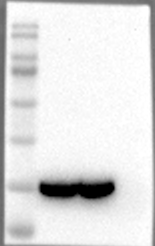

Supplement: Supplemental Information 1 — The original image of WB. [file peerj-11-16316-s001.zip › Original Images for Blots Gels/Figure3/Fig3F-GAPDH.Tif]

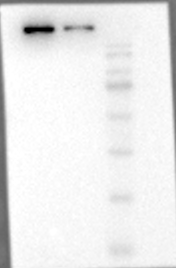

Supplement: Supplemental Information 1 — The original image of WB. [file peerj-11-16316-s001.zip › Original Images for Blots Gels/Figure3/Fig3F-ki67.Tif]

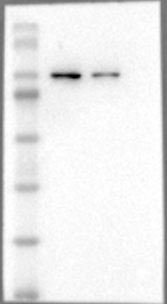

Supplement: Supplemental Information 1 — The original image of WB. [file peerj-11-16316-s001.zip › Original Images for Blots Gels/Figure3/Fig3F-MMP9.Tif]

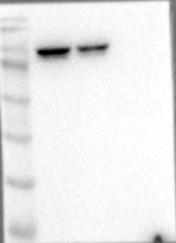

Supplement: Supplemental Information 1 — The original image of WB. [file peerj-11-16316-s001.zip › Original Images for Blots Gels/Figure3/Fig3F-p-SATA3.Tif]

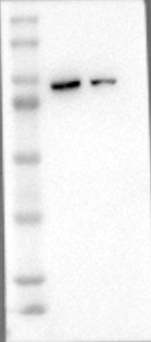

Supplement: Supplemental Information 1 — The original image of WB. [file peerj-11-16316-s001.zip › Original Images for Blots Gels/Figure3/Fig3F-TTC13.Tif]

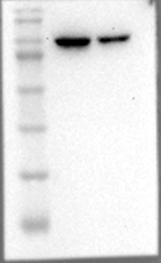

Supplement: Supplemental Information 1 — The original image of WB. [file peerj-11-16316-s001.zip › Original Images for Blots Gels/Figure3/Fig3F-a┬-catenin.Tif]

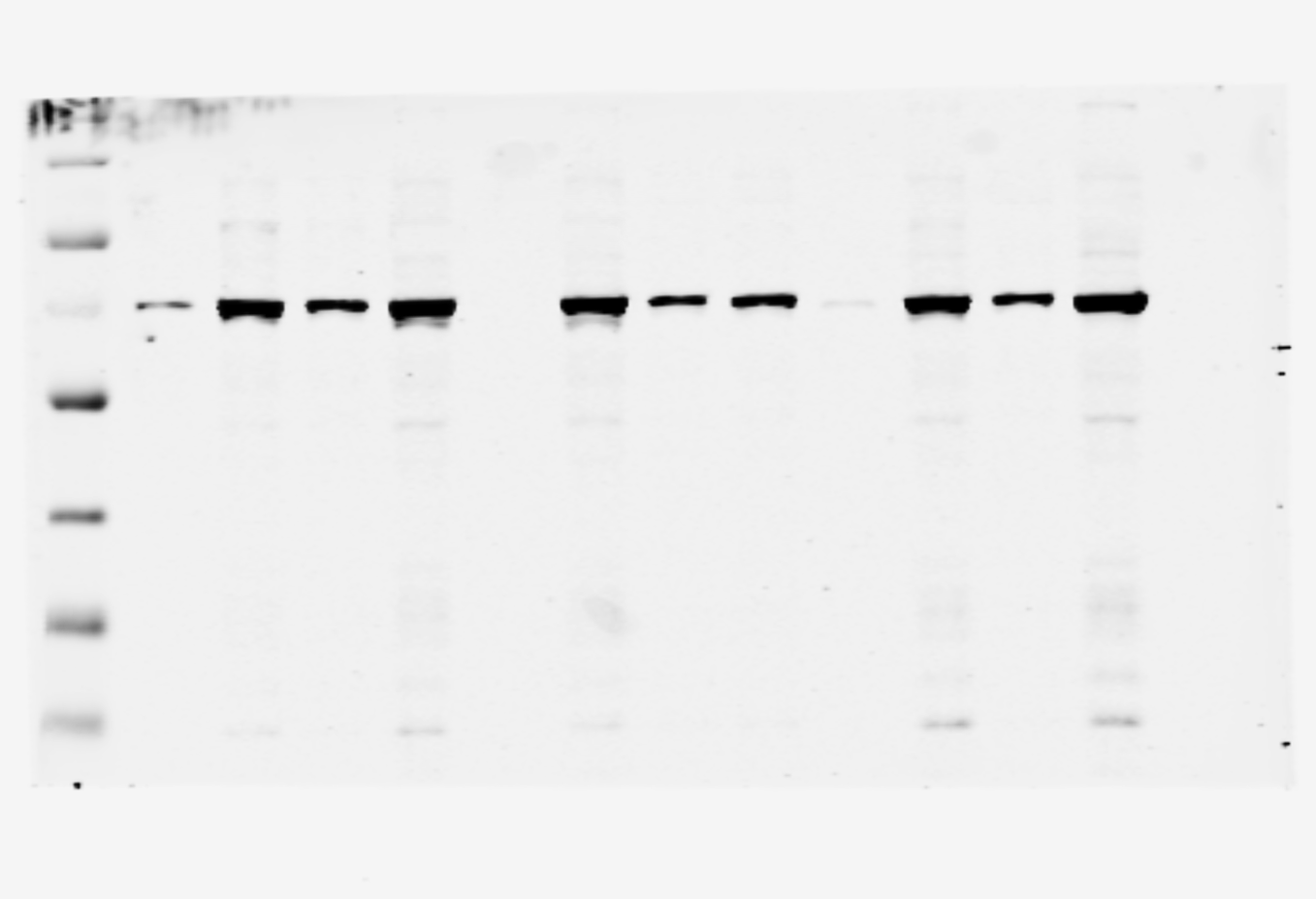

Supplement: Supplemental Information 1 — The original image of WB. [file peerj-11-16316-s001.zip › Original Images for Blots Gels/Figure4/F4-B/F4B-catenin.tif]

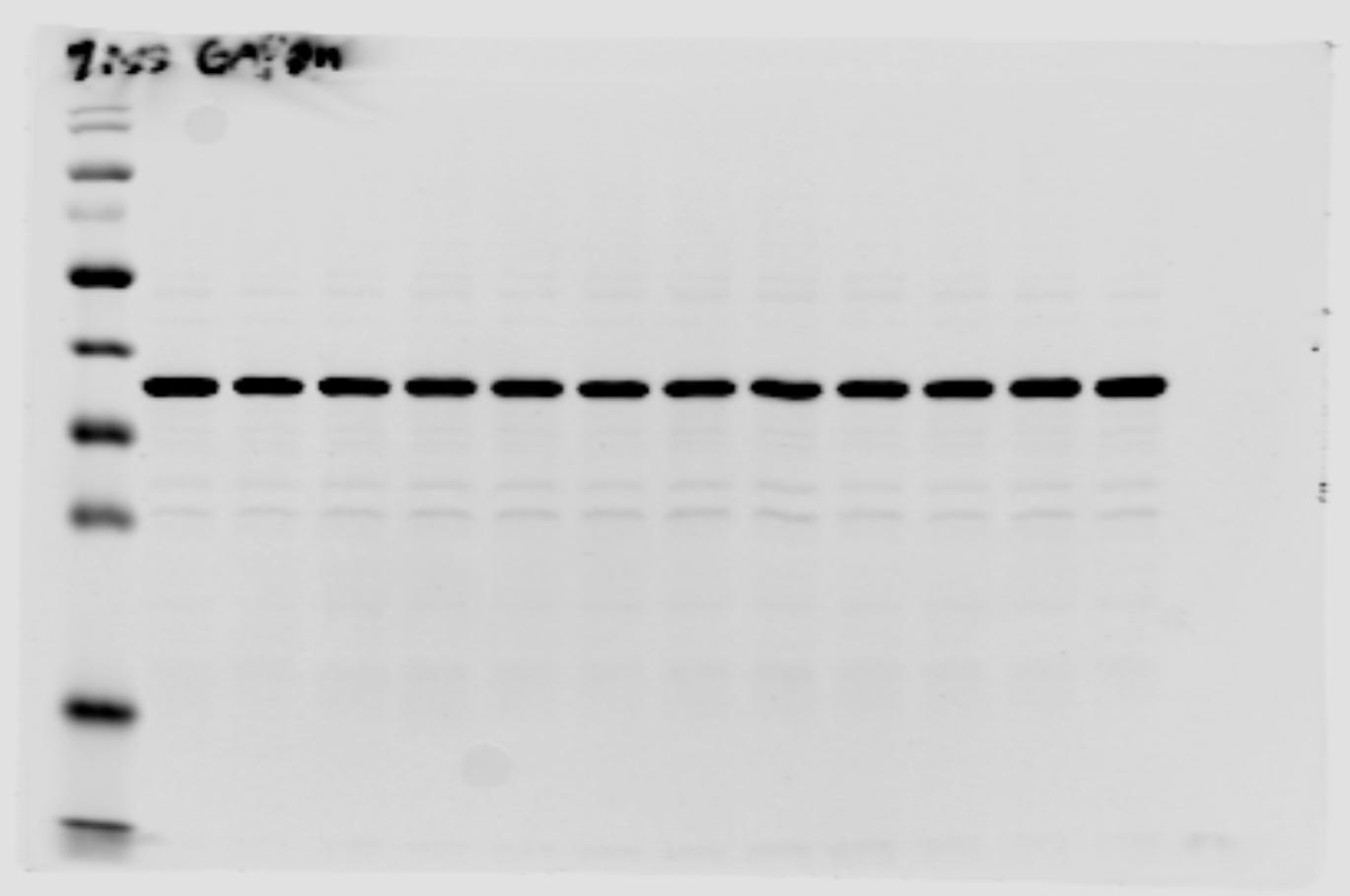

Supplement: Supplemental Information 1 — The original image of WB. [file peerj-11-16316-s001.zip › Original Images for Blots Gels/Figure4/F4-B/F4B-gapdh.tif]

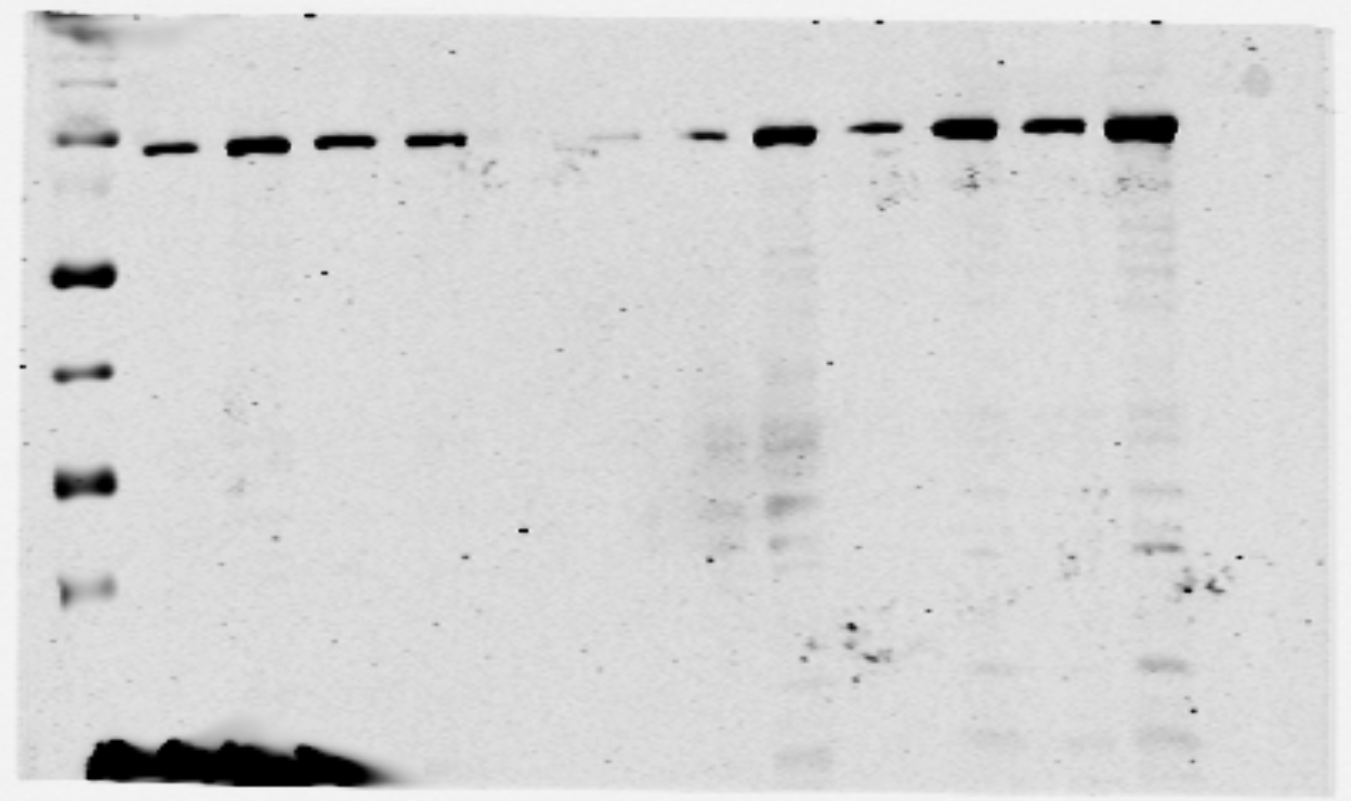

Supplement: Supplemental Information 1 — The original image of WB. [file peerj-11-16316-s001.zip › Original Images for Blots Gels/Figure4/F4-B/F4B-TTC13.tif]

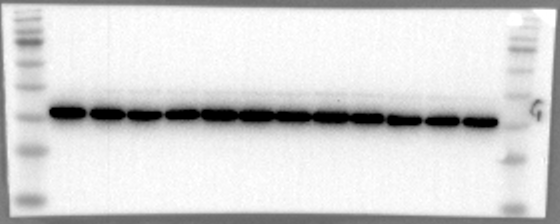

Supplement: Supplemental Information 1 — The original image of WB. [file peerj-11-16316-s001.zip › Original Images for Blots Gels/Figure4/F4-C/F4-C-GAPDH.Tif]

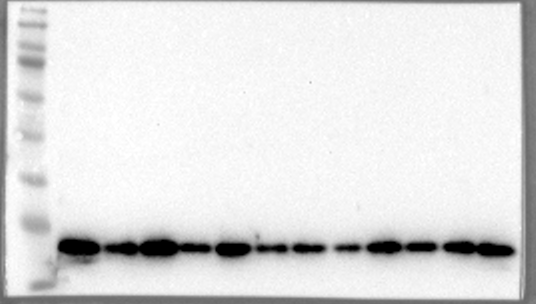

Supplement: Supplemental Information 1 — The original image of WB. [file peerj-11-16316-s001.zip › Original Images for Blots Gels/Figure4/F4-C/F4-C-IL-6.tif]

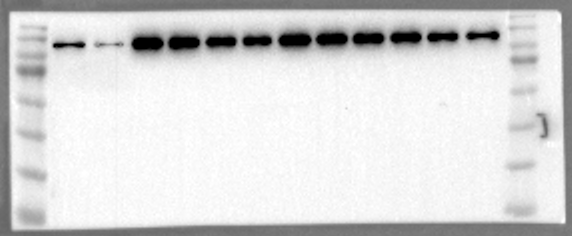

Supplement: Supplemental Information 1 — The original image of WB. [file peerj-11-16316-s001.zip › Original Images for Blots Gels/Figure4/F4-C/F4-C-JAK2.Tif]

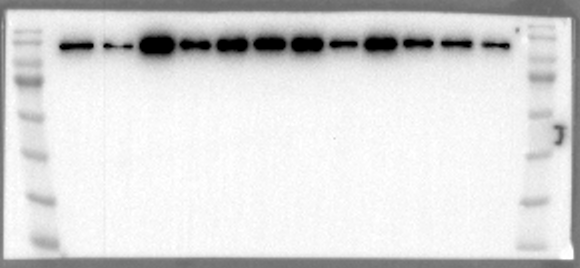

Supplement: Supplemental Information 1 — The original image of WB. [file peerj-11-16316-s001.zip › Original Images for Blots Gels/Figure4/F4-C/F4-C-P-JAK2.Tif]

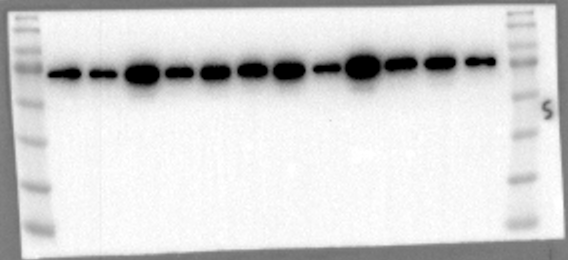

Supplement: Supplemental Information 1 — The original image of WB. [file peerj-11-16316-s001.zip › Original Images for Blots Gels/Figure4/F4-C/F4-C-P-STAT3.Tif]

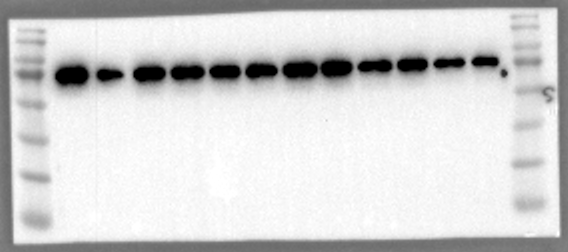

Supplement: Supplemental Information 1 — The original image of WB. [file peerj-11-16316-s001.zip › Original Images for Blots Gels/Figure4/F4-C/F4-C-STAT3.Tif]

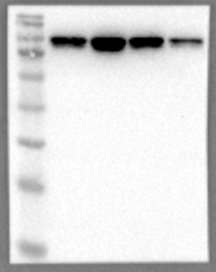

Supplement: Supplemental Information 1 — The original image of WB. [file peerj-11-16316-s001.zip › Original Images for Blots Gels/Figure4/F4-D/F4-D-786-0/F4-D-CATENIN-786-0.Tif]

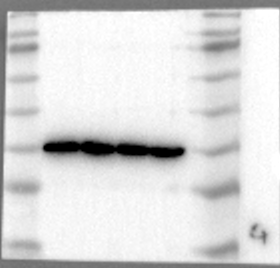

Supplement: Supplemental Information 1 — The original image of WB. [file peerj-11-16316-s001.zip › Original Images for Blots Gels/Figure4/F4-D/F4-D-786-0/F4-D-GAPDH-786-0.Tif]

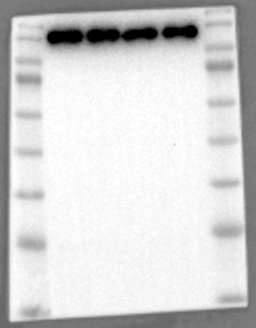

Supplement: Supplemental Information 1 — The original image of WB. [file peerj-11-16316-s001.zip › Original Images for Blots Gels/Figure4/F4-D/F4-D-786-0/F4-D-JAK2-786-0.Tif]

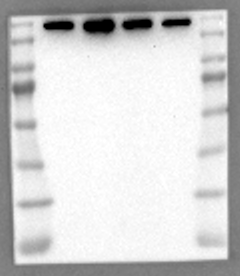

Supplement: Supplemental Information 1 — The original image of WB. [file peerj-11-16316-s001.zip › Original Images for Blots Gels/Figure4/F4-D/F4-D-786-0/F4-D-P-JAK2-786-0.Tif]

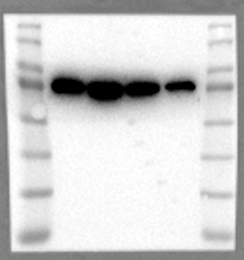

Supplement: Supplemental Information 1 — The original image of WB. [file peerj-11-16316-s001.zip › Original Images for Blots Gels/Figure4/F4-D/F4-D-786-0/F4-D-P-STAT3-786-0.Tif]

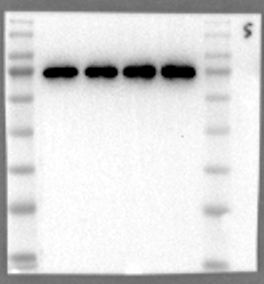

Supplement: Supplemental Information 1 — The original image of WB. [file peerj-11-16316-s001.zip › Original Images for Blots Gels/Figure4/F4-D/F4-D-786-0/F4-D-STAT3-786-0.Tif]

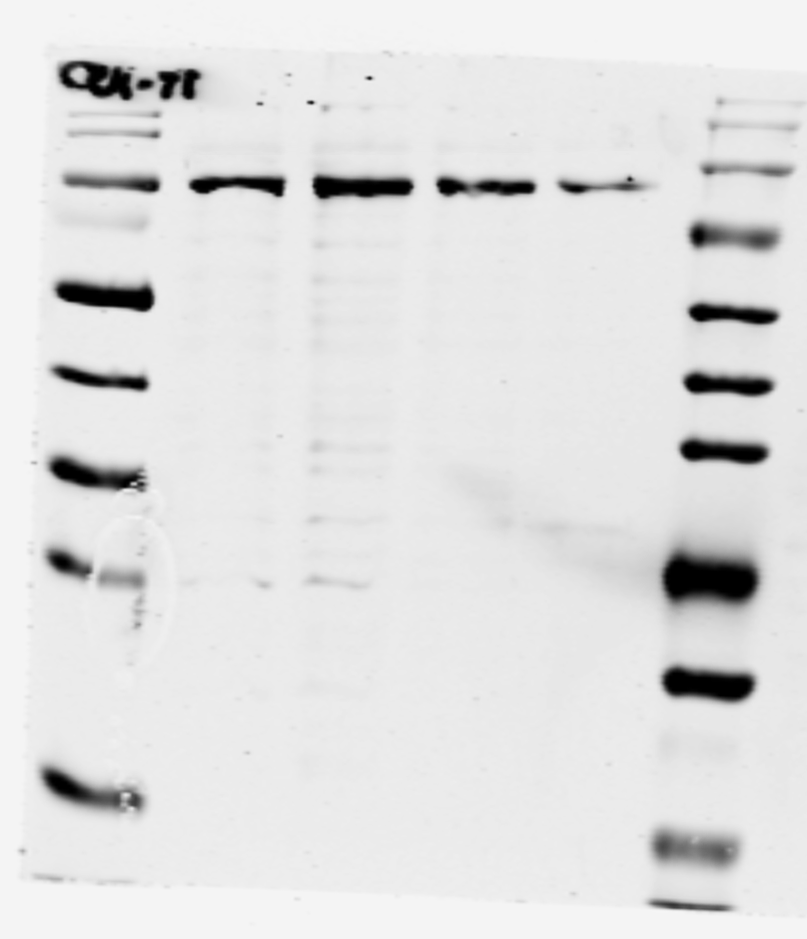

Supplement: Supplemental Information 1 — The original image of WB. [file peerj-11-16316-s001.zip › Original Images for Blots Gels/Figure4/F4-D/F4-D-786-0/F4-D-TTC13-786-0.tif]

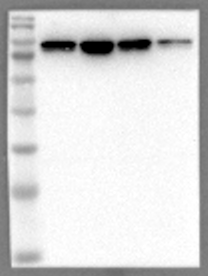

Supplement: Supplemental Information 1 — The original image of WB. [file peerj-11-16316-s001.zip › Original Images for Blots Gels/Figure4/F4-D/F4-D-CAKI-1/F4-D-CATENIN-CAKI-1.Tif]

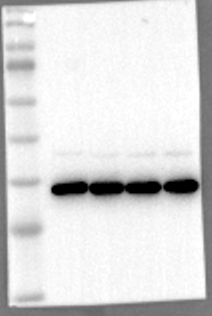

Supplement: Supplemental Information 1 — The original image of WB. [file peerj-11-16316-s001.zip › Original Images for Blots Gels/Figure4/F4-D/F4-D-CAKI-1/F4-D-GAPDH-CAKI-1.Tif]

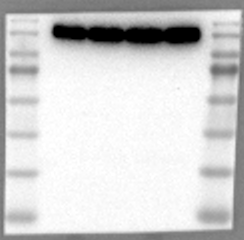

Supplement: Supplemental Information 1 — The original image of WB. [file peerj-11-16316-s001.zip › Original Images for Blots Gels/Figure4/F4-D/F4-D-CAKI-1/F4-D-JAK2-CAKI-1.Tif]

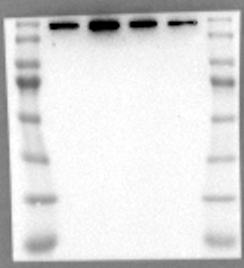

Supplement: Supplemental Information 1 — The original image of WB. [file peerj-11-16316-s001.zip › Original Images for Blots Gels/Figure4/F4-D/F4-D-CAKI-1/F4-D-P-JAK2-CAKI-1.Tif]

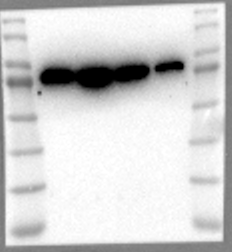

Supplement: Supplemental Information 1 — The original image of WB. [file peerj-11-16316-s001.zip › Original Images for Blots Gels/Figure4/F4-D/F4-D-CAKI-1/F4-D-P-STAT3-CAKI-1.Tif]

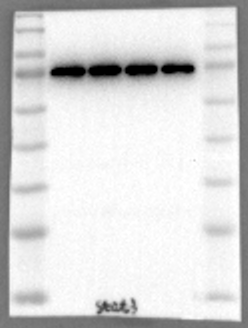

Supplement: Supplemental Information 1 — The original image of WB. [file peerj-11-16316-s001.zip › Original Images for Blots Gels/Figure4/F4-D/F4-D-CAKI-1/F4-D-STAT3-CAKI-1.Tif]

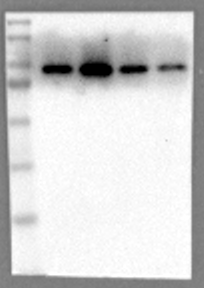

Supplement: Supplemental Information 1 — The original image of WB. [file peerj-11-16316-s001.zip › Original Images for Blots Gels/Figure4/F4-D/F4-D-CAKI-1/F4-D-TTC13-CAKI-1.Tif]

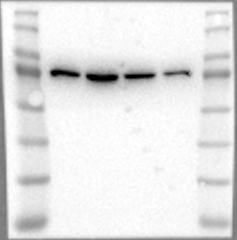

Supplement: Supplemental Information 1 — The original image of WB. [file peerj-11-16316-s001.zip › Original Images for Blots Gels/Figure4/F4-D/F4-D-p-stat3/Fig4D Caki-1 p-stat3.Tif]

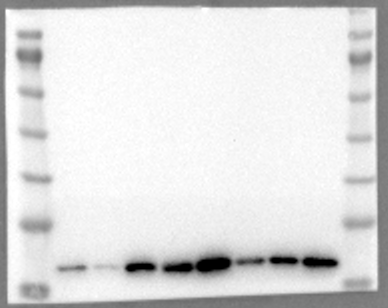

Supplement: Supplemental Information 1 — The original image of WB. [file peerj-11-16316-s001.zip › Original Images for Blots Gels/Figure5/F5-786-0/F5-BAX.Tif]

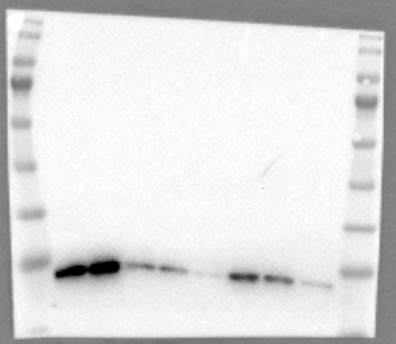

Supplement: Supplemental Information 1 — The original image of WB. [file peerj-11-16316-s001.zip › Original Images for Blots Gels/Figure5/F5-786-0/F5-BCL-2.Tif]

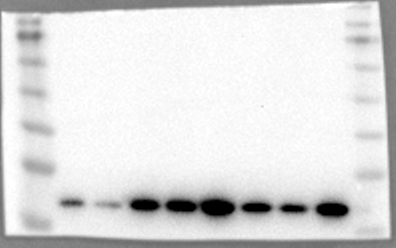

Supplement: Supplemental Information 1 — The original image of WB. [file peerj-11-16316-s001.zip › Original Images for Blots Gels/Figure5/F5-786-0/F5-CASPASE3.Tif]

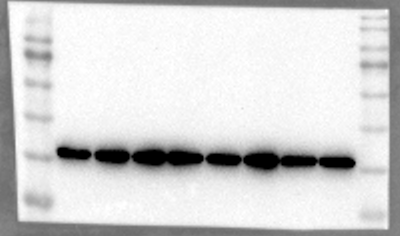

Supplement: Supplemental Information 1 — The original image of WB. [file peerj-11-16316-s001.zip › Original Images for Blots Gels/Figure5/F5-786-0/F5-GAPDH.Tif]

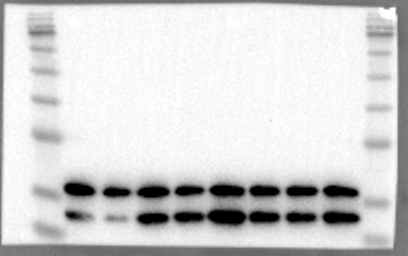

Supplement: Supplemental Information 1 — The original image of WB. [file peerj-11-16316-s001.zip › Original Images for Blots Gels/Figure5/F5-786-0/F5-LC3.Tif]

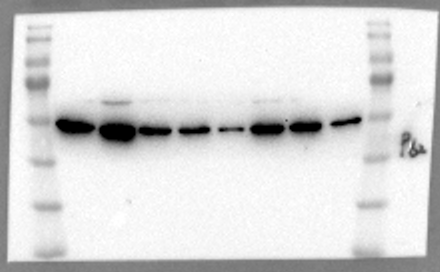

Supplement: Supplemental Information 1 — The original image of WB. [file peerj-11-16316-s001.zip › Original Images for Blots Gels/Figure5/F5-786-0/F5-P62.Tif]

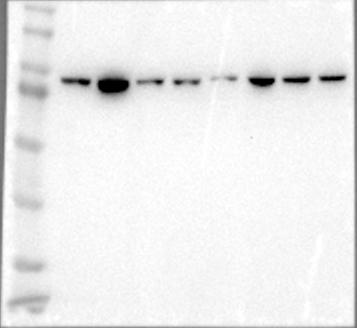

Supplement: Supplemental Information 1 — The original image of WB. [file peerj-11-16316-s001.zip › Original Images for Blots Gels/Figure5/F5-786-0/Fig5D-786-0-TTC13.Tif]

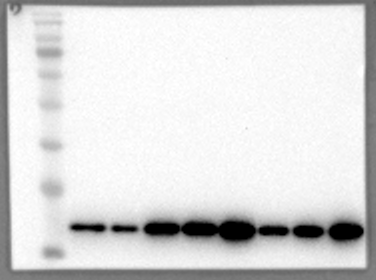

Supplement: Supplemental Information 1 — The original image of WB. [file peerj-11-16316-s001.zip › Original Images for Blots Gels/Figure5/F5-CAKI-1/F5-BAX.Tif]

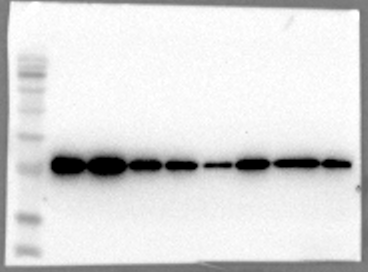

Supplement: Supplemental Information 1 — The original image of WB. [file peerj-11-16316-s001.zip › Original Images for Blots Gels/Figure5/F5-CAKI-1/F5-BCL-2.Tif]

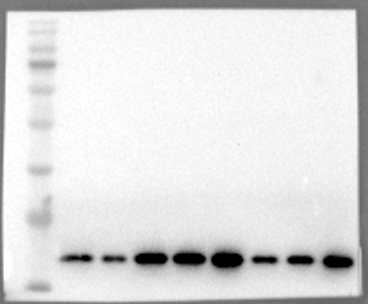

Supplement: Supplemental Information 1 — The original image of WB. [file peerj-11-16316-s001.zip › Original Images for Blots Gels/Figure5/F5-CAKI-1/F5-CASPASE3.Tif]

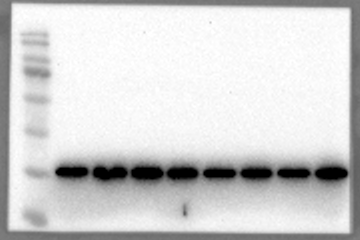

Supplement: Supplemental Information 1 — The original image of WB. [file peerj-11-16316-s001.zip › Original Images for Blots Gels/Figure5/F5-CAKI-1/F5-GAPDH.Tif]

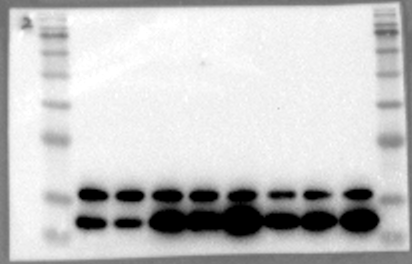

Supplement: Supplemental Information 1 — The original image of WB. [file peerj-11-16316-s001.zip › Original Images for Blots Gels/Figure5/F5-CAKI-1/F5-LC3.Tif]

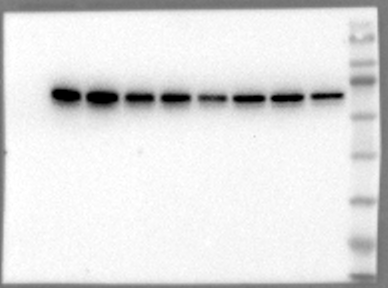

Supplement: Supplemental Information 1 — The original image of WB. [file peerj-11-16316-s001.zip › Original Images for Blots Gels/Figure5/F5-CAKI-1/F5-P62.Tif]

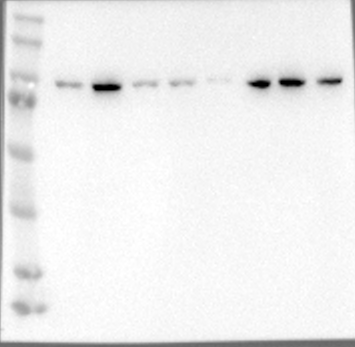

Supplement: Supplemental Information 1 — The original image of WB. [file peerj-11-16316-s001.zip › Original Images for Blots Gels/Figure5/F5-CAKI-1/Fig5D-Caki-1-TTC13.Tif]

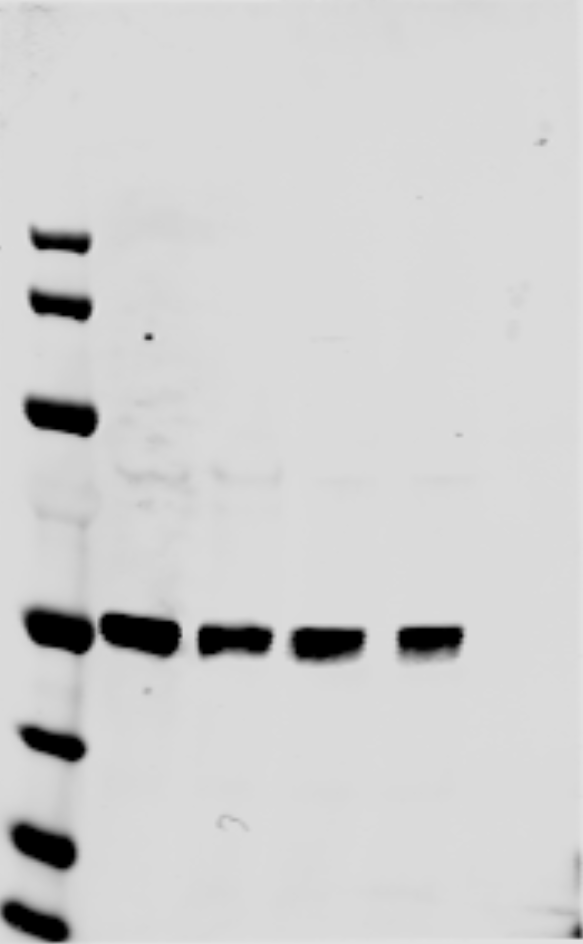

Supplement: Supplemental Information 1 — The original image of WB. [file peerj-11-16316-s001.zip › Original Images for Blots Gels/Figure6/Fig6C-786-0-GAPDH.tif]

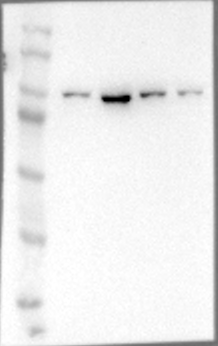

Supplement: Supplemental Information 1 — The original image of WB. [file peerj-11-16316-s001.zip › Original Images for Blots Gels/Figure6/Fig6C-786-0-TTC13.Tif]

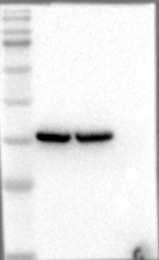

Supplement: Supplemental Information 1 — The original image of WB. [file peerj-11-16316-s001.zip › Original Images for Blots Gels/Figure7s/Fig7S-786-0-GAPDH.Tif]

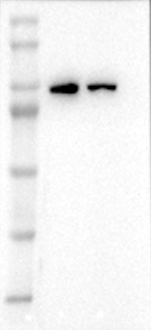

Supplement: Supplemental Information 1 — The original image of WB. [file peerj-11-16316-s001.zip › Original Images for Blots Gels/Figure7s/Fig7S-786-0-TTC13.Tif]

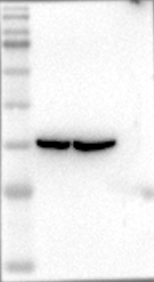

Supplement: Supplemental Information 1 — The original image of WB. [file peerj-11-16316-s001.zip › Original Images for Blots Gels/Figure7s/Fig7S-tumor-GAPDH.Tif]

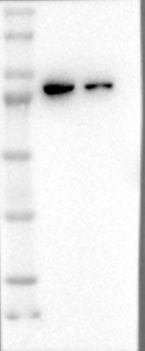

Supplement: Supplemental Information 1 — The original image of WB. [file peerj-11-16316-s001.zip › Original Images for Blots Gels/Figure7s/Fig7S-tumor-TTC13.Tif]

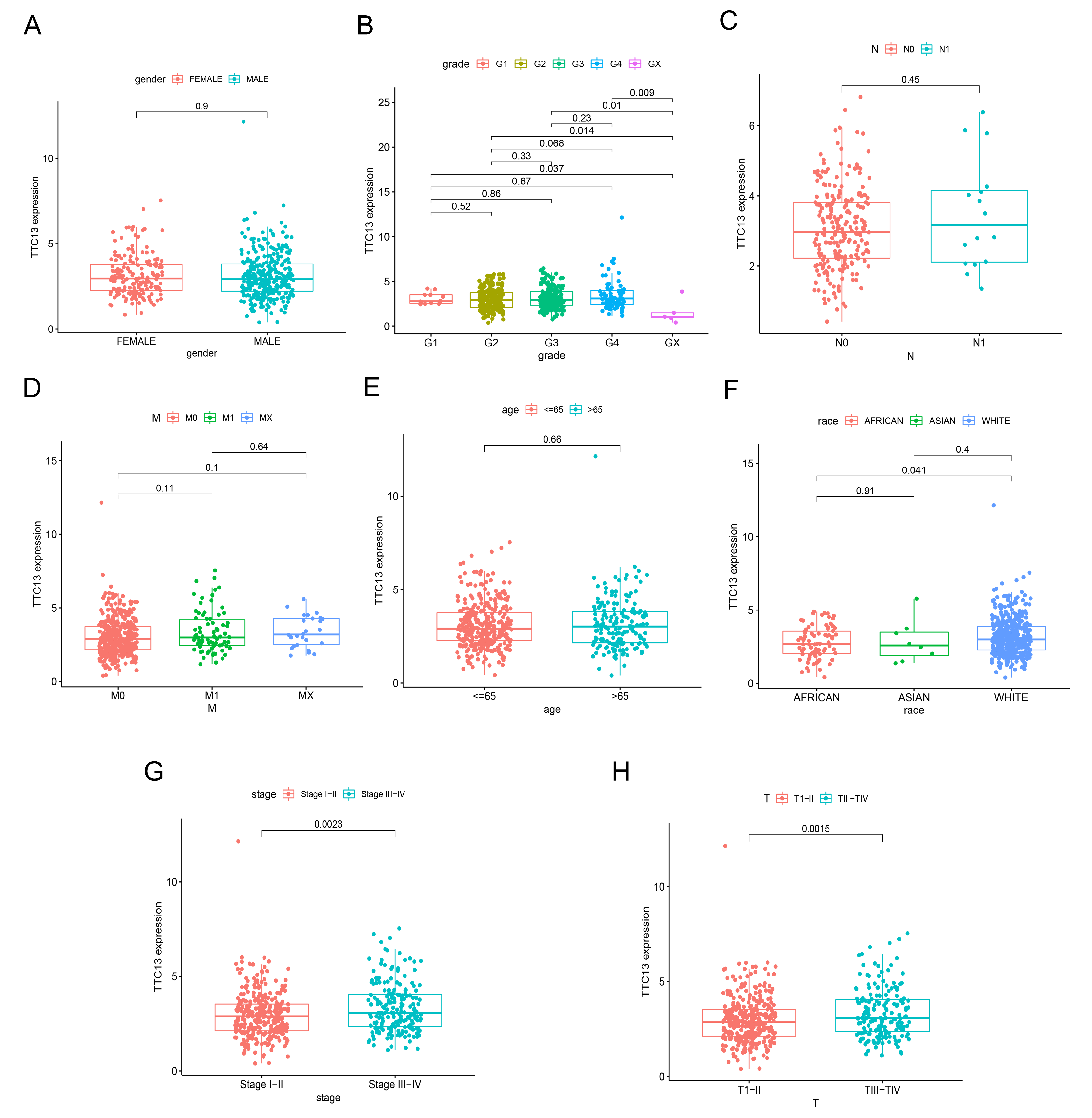

Supplement: Figure 1S [file peerj-11-16316-s004.tif]

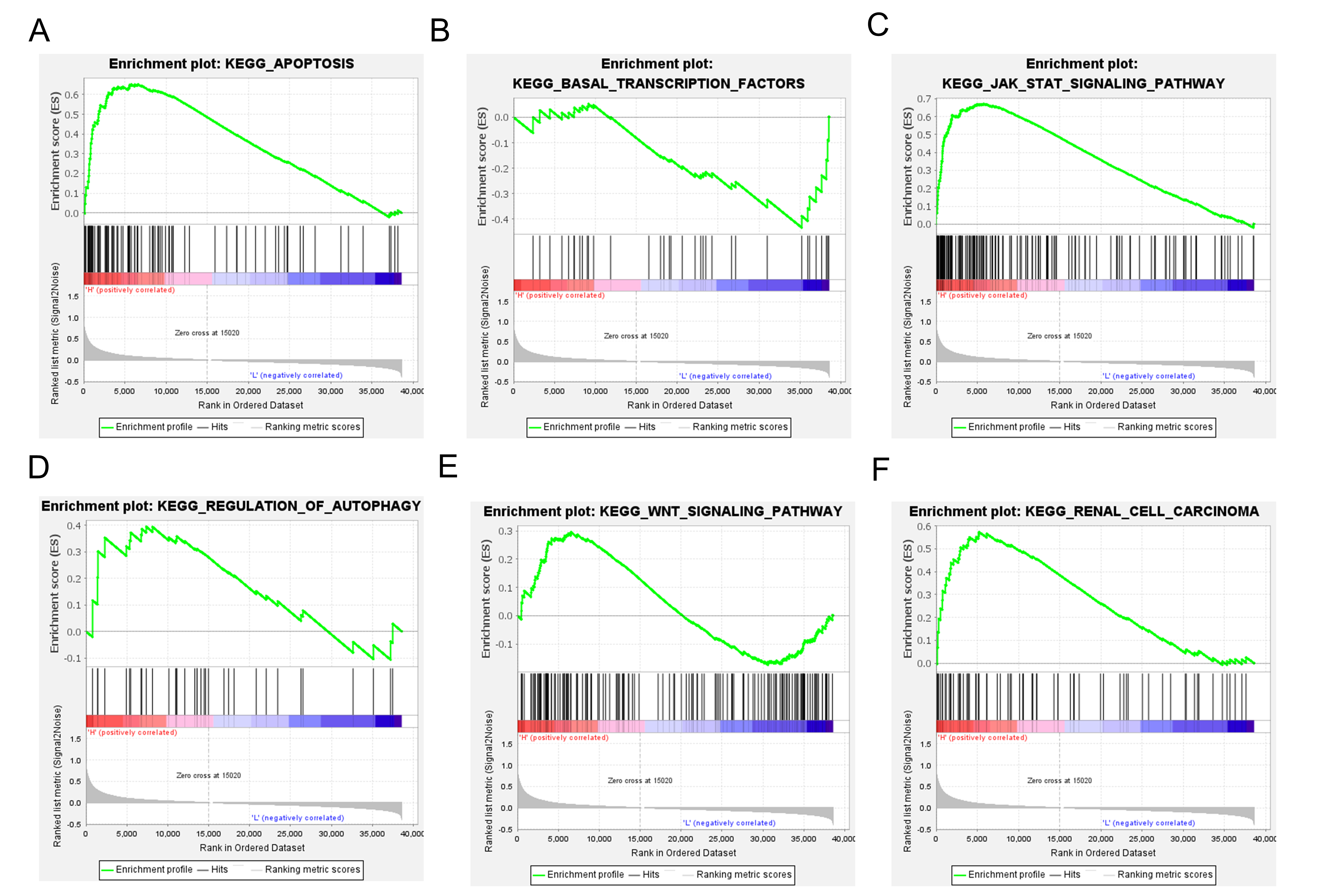

Supplement: Figure 2S [file peerj-11-16316-s005.tif]

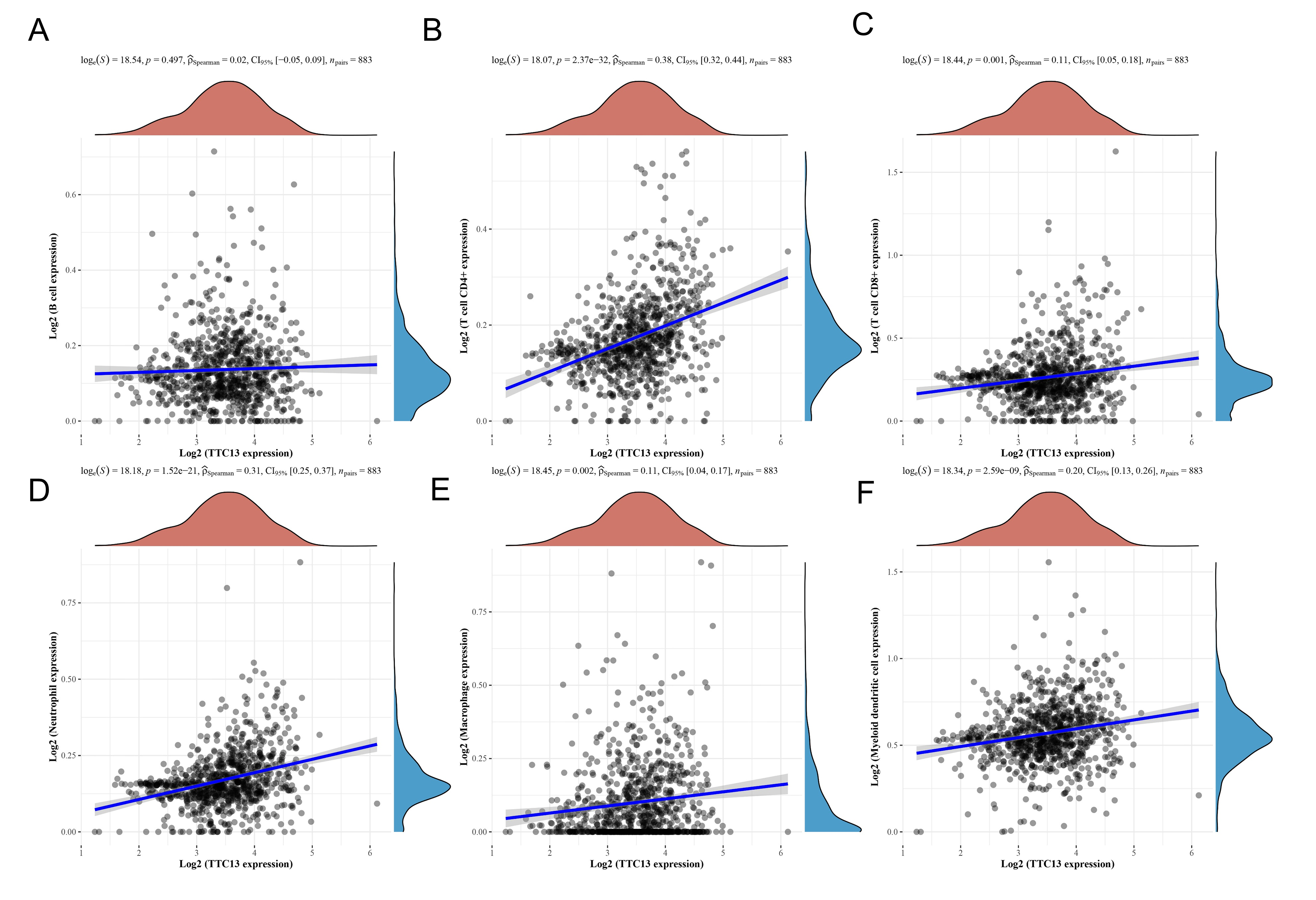

Supplement: Figure 3S [file peerj-11-16316-s006.tif]

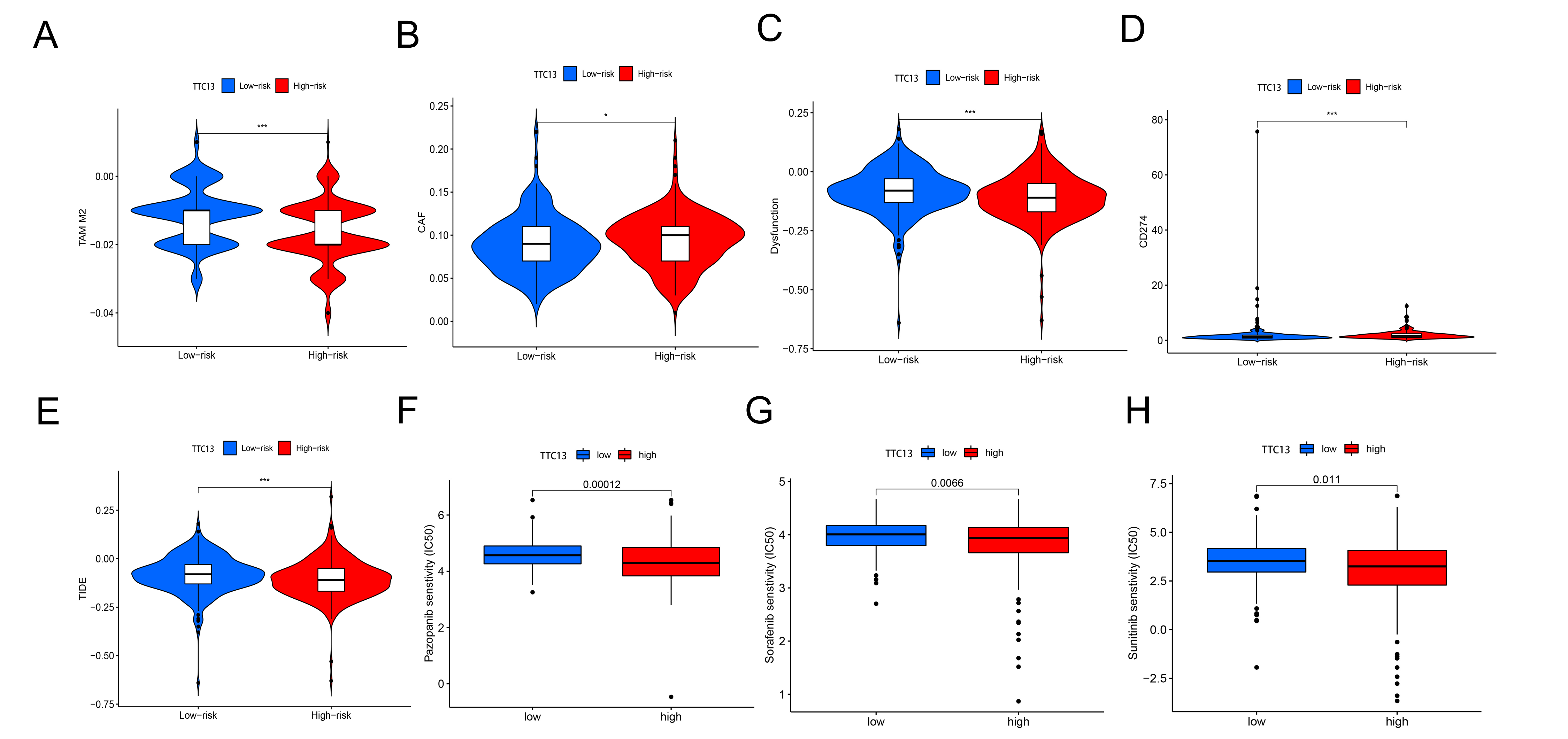

Supplement: Figure 4S [file peerj-11-16316-s007.tif]

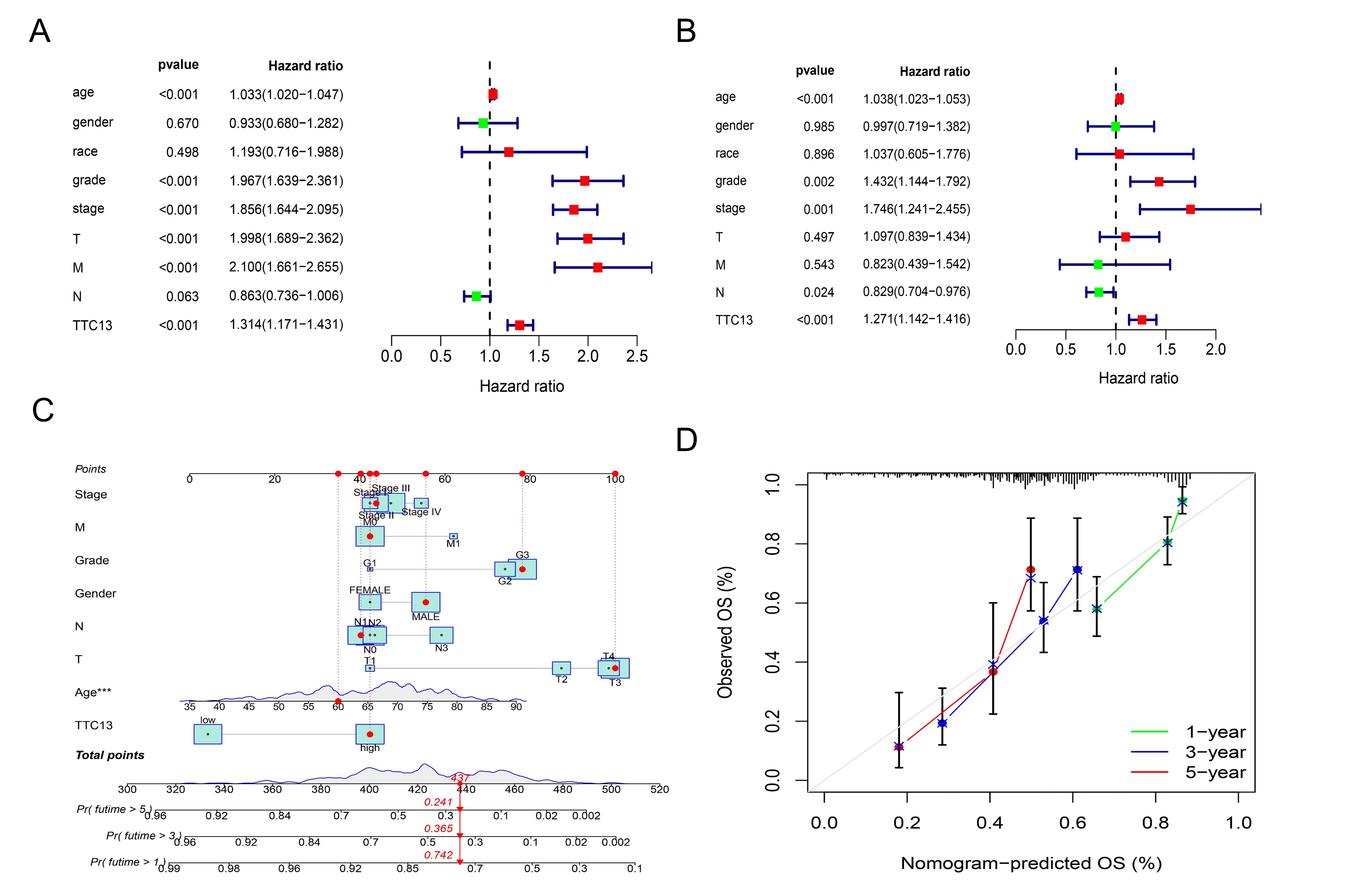

Supplement: Figure 5S [file peerj-11-16316-s008.tif]

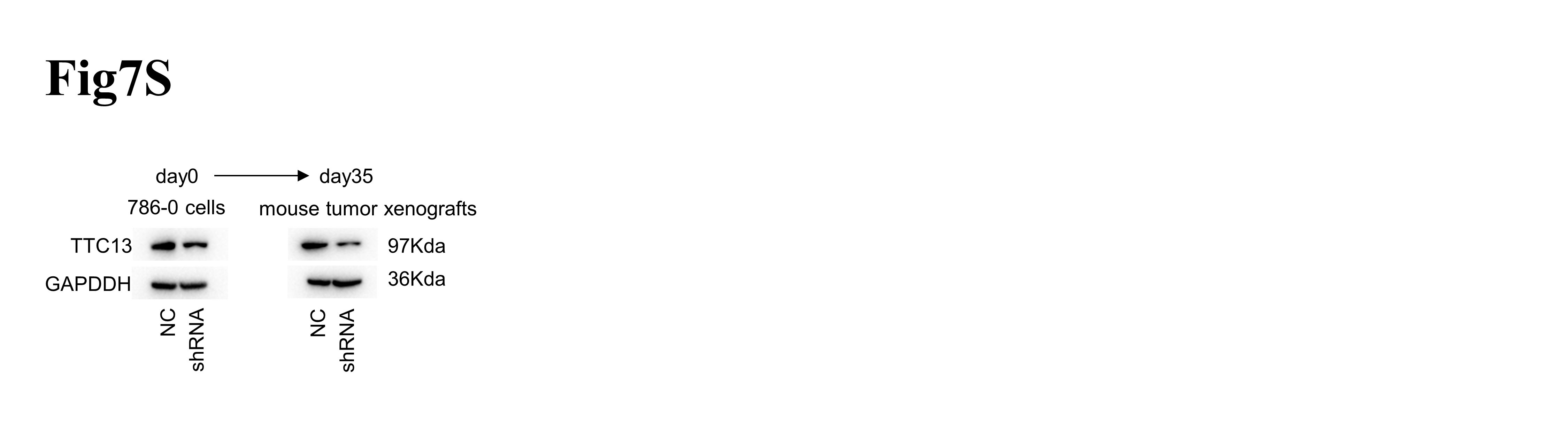

Supplement: Figure 6S [file peerj-11-16316-s009.jpg]
