# Supplementary material for: TTC13 expression and STAT3 activation may form a positive feedback loop to promote ccRCC progression
Source: PeerJ. 2023 Oct 30;11:e16316. doi: 10.7717/peerj.16316 (PMC10621595; doi:10.7717/peerj.16316)
Supplement: Supplemental Information 2 [file peerj-11-16316-s002.docx]

#### download TCGA expression profile data ####

library(SummarizedExperiment)

library(TCGAbiolinks)

query <- GDCquery(project = "TCGA-KIRC",

data.category = "Transcriptome Profiling",

data.type = "Gene Expression Quantification",

workflow.type = "STAR - Counts",

legacy = FALSE)

# Expand memory

memory.limit(1000000)

GDCdownload(query = query)

mydata=GDCprepare(query = query)

mydata2=as.data.frame(rowRanges(mydata))

geneexp <- assay(mydata,i = "tpm_unstrand")#tpm_unstrand fpkm_unstrand #unstranded

geneexp=as.data.frame(geneexp)

geneexp1=cbind(gene_type=mydata2$gene_type,gene_name=mydata2$gene_name,geneexp)

mRNA=geneexp1[geneexp1$gene_type=="protein_coding",]

mRNA=mRNA[,-1]

TPM <- mRNA[!duplicated(mRNA$gene_name),]

rownames(TPM) <- TPM$gene_name

TPM <- TPM[,-1]

TPM <- TPM[,substr(colnames(TPM),14,16)%in% c("01A","11A")]

### Determine if log2 is present

# Automatic logging (x)

ex <- TPM

qx <- as.numeric(quantile(ex, c(0., 0.25, 0.5, 0.75, 0.99, 1.0), na.rm=T))

LogC <- (qx[5] > 100) ||

(qx[6]-qx[1] > 50 && qx[2] > 0) ||

(qx[2] > 0 && qx[2] < 1 && qx[4] > 1 && qx[4] < 2)

if (LogC) {

ex[which(ex < 0,arr.ind=T)] <- NaN # Define negative numbers as NaN

exprSet <- log2(ex+1) #log2(x+1)

print("log2 transform finished")

}else{

print("log2 transform not needed")

}

# Remove NaN

exprSet[which(exprSet == "NaN",arr.ind=T)]

expr_TCGA_TPM <- as.data.frame(exprSet)

tumor <- colnames(expr_TCGA_TPM)[substr(colnames(expr_TCGA_TPM),14,16) == "01A"] #533

normal <- colnames(expr_TCGA_TPM)[substr(colnames(expr_TCGA_TPM),14,16) == "11A"] #72

expr_TCGA_TPM <- expr_TCGA_TPM[,c(normal,tumor)]

expr_TCGA_TPM_01A <- expr_TCGA_TPM[,tumor]

expr_TCGA_TPM_11A <- expr_TCGA_TPM[,normal]

save(expr_TCGA_TPM,expr_TCGA_TPM_01A,expr_TCGA_TPM_11A,file="data/TCGA_KIRC_mRNA.Rdata")

# end #

#### Download TCGA Clinical Data ####

rm(list = ls())

library(dplyr)

library(tibble)

library(tidyr)

library(TCGAbiolinks)

options(stringsAsFactors = F)

## 1. Clinical information download

clinical <- GDCquery_clinic(project = "TCGA-KIRC",

type = "clinical")

dput(colnames(clinical)) # Magical operation to directly generate vectors

clin_data <- clinical %>%

dplyr::select(patient_ID = submitter_id,

vital_status,

gender,

age = age_at_diagnosis,

Stage = ajcc_pathologic_stage,

T_Stage = ajcc_pathologic_t ,

N_Stage = ajcc_pathologic_n,

M_Stage = ajcc_pathologic_m,

days_to_death,

days_to_last_follow_up)

clin_data$age <- (as.numeric(clin_data$age))/365

clin_data$days_to_death <- as.numeric(clin_data$days_to_death)

clin_data$days_to_last_follow_up <- as.numeric(clin_data$days_to_last_follow_up)

# Add time and status, remove empty values from these two columns

rt <- clin_data %>%

mutate(time = coalesce(days_to_death, days_to_last_follow_up)/365,

status = ifelse(vital_status == 'Alive', 0, 1)) %>%

dplyr::filter(time > 0) %>%

dplyr::filter(age > 0) %>%

distinct() %>%

dplyr::select(- c("days_to_death", "days_to_last_follow_up"))

rt <- rt[-which(rt$N_Stage == "NX"),]

rt <- rt[-which(rt$M_Stage == "MX"),]

rt$gender <- factor(rt$gender,labels=c("female", "male"))

rt$Stage <- if_else(rt$Stage %in% c("Stage IV"),"Stage IV",

if_else(rt$Stage %in% c("Stage III"),"Stage III",

if_else(rt$Stage %in% c("Stage II"),"Stage II",

"Stage I")))

rt$Stage <- factor(rt$Stage,labels=c("Stage I", "Stage II", "Stage III", "Stage IV"))

rt$T_Stage <- if_else(rt$T_Stage %in% c("T1","T1a","T1b"),"T1",

if_else(rt$T_Stage %in% c("T2","T2a","T2b"),"T2",

if_else(rt$T_Stage%in% c("T3","T3a","T3b","T3c"),"T3","T4")))

rt$T_Stage <- factor(rt$T_Stage,labels=c("T1", "T2", "T3", "T4"))

rt$N_Stage <- factor(rt$N_Stage,labels=c("N0", "N1"))

rt$M_Stage <- factor(rt$M_Stage,labels=c("M0", "M1"))

TCGA_clinical <- rt

save(TCGA_clinical,file = "data/TCGA_KIRC_clinic.Rdata")

# end #

#### TTC13 unpaired sample expression level ####

load(file = "data/TCGA_KIRC_mRNA.Rdata")

exprSet <- expr_TCGA_TPM["TTC13",]

TCGA_id <- colnames(exprSet)

sample <- ifelse(substring(TCGA_id,14,15)=="01","Tumor","Normal")

sample <- factor(sample,levels = c("Normal","Tumor")) # The purpose of factor is to rank normal first

metadata <- data.frame(TCGA_id,sample)

exprSet <-t(exprSet)

exprSet <-as.data.frame(exprSet)

exprSet <- cbind(TCGA_id=rownames(exprSet),exprSet) # The row name cannot be directly changed to the first column, it must be cbind

exprSet <- merge(metadata,exprSet,by="TCGA_id")

# draw designs

library(ggplot2)

library(ggpubr)

# Set the comparison group for p

compare_means(TTC13 ~ sample, data = exprSet) # Gene, sample, data

p <- ggboxplot(exprSet, x = "sample", y = "TTC13",

color = "sample", palette = "jco",

add = "jitter")+ylab("The expression of TTC13")

p + stat_compare_means( aes(label = ..p.signif..),

label.x = 1.5, label.y = 6)

#### TTC13 paired sample expression level ####

library(limma)

load(file = "data/TCGA_KIRC_mRNA.Rdata")

exprSet <- expr_TCGA_TPM["TTC13",]

TCGA_id <- colnames(exprSet)

sample <- ifelse(substring(TCGA_id,14,15)=="01","Tumor","Normal")

sample <- factor(sample,levels = c("Normal","Tumor")) # The purpose of factor is to rank normal first

metadata <- data.frame(TCGA_id,sample)

exprSet <-t(exprSet)

exprSet <-as.data.frame(exprSet)

exprSet <- cbind(TCGA_id=rownames(exprSet),exprSet) # The row name cannot be directly changed to the first column, it must be cbind

exprSet <- merge(metadata,exprSet,by="TCGA_id")

geneName="TTC13"

# Extract data from paired samples

rownames(exprSet) <- exprSet$TCGA_id

normalData=exprSet[exprSet$sample=="Normal",3,drop=F]

normalData=as.matrix(normalData)

rownames(normalData)=gsub("(.*?)\\-(.*?)\\-(.*?)\\-(.*?)\\-.*", "\\1\\-\\2\\-\\3", rownames(normalData))

normalData=avereps(normalData)

tumorData=exprSet[exprSet$sample=="Tumor",3,drop=F]

tumorData=as.matrix(tumorData)

rownames(tumorData)=gsub("(.*?)\\-(.*?)\\-(.*?)\\-(.*?)\\-.*", "\\1\\-\\2\\-\\3", rownames(tumorData))

tumorData=avereps(tumorData)

sameSample=intersect(row.names(normalData), row.names(tumorData))

data=cbind(normalData[sameSample,,drop=F], tumorData[sameSample,,drop=F])

colnames(data)=c("Normal", "Tumor")

data=as.data.frame(data)

library(ggplot2)

library(ggpubr)

# Draw a graph for paired difference analysis

ggpaired(data, cond1="Normal", cond2="Tumor", fill="condition",

xlab="", ylab=paste0(geneName, " expression"),

legend.title="Type",

color = "condition", line.color = "gray", line.size = 0.4,

palette = "jco")+

stat_compare_means(paired = TRUE, symnum.args=list(cutpoints = c(0, 0.001, 0.01, 0.05, 1), symbols = c("***", "**", "*", "ns")),label = "p.signif",label.x = 1.35)

dev.off()

#### Prognosis KM ####

load(file = "data/TCGA_KIRC_mRNA.Rdata")

load(file = "data/TCGA_KIRC_clinic.Rdata")

colnames(expr_TCGA_TPM_01A) <- substr(colnames(expr_TCGA_TPM_01A),1,12)

expr_TCGA_TPM_01A <- expr_TCGA_TPM_01A["TTC13",]

rownames(TCGA_clinical) <- TCGA_clinical$patient_ID

comgene <- intersect(rownames(TCGA_clinical),colnames(expr_TCGA_TPM_01A))

TCGA_clinical <- TCGA_clinical[comgene,]

expr_TCGA_TPM_01A <- expr_TCGA_TPM_01A[,comgene]

expr_TCGA_TPM_01A <- as.data.frame(expr_TCGA_TPM_01A)

expr_TCGA_TPM_01A <- t(expr_TCGA_TPM_01A)

rt <- cbind(expr_TCGA_TPM_01A,TCGA_clinical)

save(rt,file = "data/TTC13_clinic.Rdata")

library(survival)

library(survminer)

res.cut <- surv_cutpoint(rt,

time ="time",

event = "status",

variables = "TTC13",

minprop = 0.3)

res.cut

cutoff=res.cut$cutpoint$cutpoint

rt$TTC13 <- ifelse(rt$TTC13 > cutoff, "TTC13_high","TTC13_low")

rt <- as.data.frame(rt)

# grouping

fit <- survfit(Surv(time, status) ~ TTC13, data = rt)

p1 <- ggsurvplot(fit,

legend.title = "",# Define the name of the legend

legend.labs = c("TTC13_high","TTC13_low"),# Be sure to match the order of the table

#legend = "top",# legend location

pval = T,

pval.method = TRUE,# adding test method for p-value

#conf.int = TRUE,# Add Confidence Interval

risk.table = TRUE, # Add a risk table below the figure

risk.table.col = "strata", # Add colors to the risk table based on data grouping

risk.table.y.text = F,# Does the Y-axis of the risk table display the name of the group, and F displays the group as a line

#linetype = "strata", # Changing the Linetype of Survival Curves for Different Groups

#surv.median.line = "hv", # Mark the median survival time

xlab = "Time in years", # X-axis title

xlim = c(0,max(rt$time)), # Show the range of the x-axis

ylab = "Overall survival rate",

break.time.by = 1, # X-axis spacing

size = 1, # Line size

#ggtheme = theme_bw(), # Adding a Grid to a Graph

palette = "jco"# Graphic Color Style

)

# Take a look at the following picture

p1

#### ROC curve ####

library(ROCR)

library(glmnet)

library(caret)

library(timeROC)

library(survival)

load(file = "data/TTC13_clinic.Rdata")

time_roc_res <- timeROC(

T=rt$time, # Ending time

delta=rt$status, # Outcome indicators

marker=rt$TTC13,

cause = 1,

weighting="marginal",

times = c(1, 3, 5), # Change time and years

ROC = TRUE,

iid = TRUE

)

time_roc_res$AUC

confint(time_roc_res, level = 0.95)$CI_AUC

time_ROC_df <- data.frame(

TP_1year = time_roc_res$TP[, 1],

FP_1year = time_roc_res$FP[, 1],

TP_2year = time_roc_res$TP[, 2],

FP_2year = time_roc_res$FP[, 2],

TP_3year = time_roc_res$TP[, 3],

FP_3year = time_roc_res$FP[, 3]

)

ggplot(data = time_ROC_df) +

geom_line(aes(x = FP_1year, y = TP_1year), size = 1, color = "#BC3C29FF") +

geom_line(aes(x = FP_2year, y = TP_2year), size = 1, color = "#0072B5FF") +

geom_line(aes(x = FP_3year, y = TP_3year), size = 1, color = "#E18727FF") +

geom_abline(slope = 1, intercept = 0, color = "grey", size = 1, linetype = 2) +

theme_bw() +

annotate("text",

x = 0.75, y = 0.25, size = 4.5,

label = paste0("AUC at 1 years = ", sprintf("%.3f", time_roc_res$AUC[[1]])), color = "#BC3C29FF" # change name

) +

annotate("text",

x = 0.75, y = 0.15, size = 4.5,

label = paste0("AUC at 3 years = ", sprintf("%.3f", time_roc_res$AUC[[2]])), color = "#0072B5FF" # change name

) +

annotate("text",

x = 0.75, y = 0.05, size = 4.5,

label = paste0("AUC at 5 years = ", sprintf("%.3f", time_roc_res$AUC[[3]])), color = "#E18727FF" # change name

) +

labs(x = "False positive rate", y = "True positive rate") +

theme(

axis.text = element_text(face = "bold", size = 11, color = "black"),

axis.title.x = element_text(face = "bold", size = 14, color = "black", margin = margin(c(15, 0, 0, 0))),

axis.title.y = element_text(face = "bold", size = 14, color = "black", margin = margin(c(0, 15, 0, 0)))

)

# complete #

#### Correlation between TTC13 and clinical features ####

load(file = "data/TTC13_clinic.Rdata")

#stage

rt$Stage <- if_else(rt$Stage %in% c("Stage I","Stage II"),"Stage I-II", "Stage III-IV")

rt$Stage <- factor(rt$Stage,labels=c("Stage I-II", "Stage III-IV"))

compare_means(TTC13 ~ Stage, data = rt, method = "anova")

p <- ggboxplot(rt, x = "Stage", y = "TTC13",

color = "Stage", palette = "jco",

add = "jitter")+ylab("The expression of TTC13")

p + stat_compare_means(label.x = 1.5, label.y = 6)

#T_Stage

rt$T_Stage <- if_else(rt$T_Stage %in% c("T1","T2"),"T1-2", "T3-4")

rt$T_Stage <- factor(rt$T_Stage,labels=c("T1-2", "T3-4"))

compare_means(TTC13 ~ T_Stage, data = rt, method = "anova")

p <- ggboxplot(rt, x = "T_Stage", y = "TTC13",

color = "T_Stage", palette = "jco",

add = "jitter")+ylab("The expression of TTC13")

p + stat_compare_means(label.x = 1.5, label.y = 6)

#N_Stage

compare_means(TTC13 ~ N_Stage, data = rt, method = "anova")

p <- ggboxplot(rt, x = "N_Stage", y = "TTC13",

color = "N_Stage", palette = "jco",

add = "jitter")+ylab("The expression of TTC13")

p + stat_compare_means(label.x = 1.5, label.y = 6)

#M_Stage

compare_means(TTC13 ~ M_Stage, data = rt, method = "anova")

p <- ggboxplot(rt, x = "M_Stage", y = "TTC13",

color = "M_Stage", palette = "jco",

add = "jitter")+ylab("The expression of TTC13")

p + stat_compare_means(label.x = 1.5, label.y = 6)

#Age

rt$age <- ifelse(rt$age >=65, ">=65","<65")

rt$age<-factor(rt$age,levels=c("<65",">=65"))

compare_means(TTC13 ~ age, data = rt, method = "anova")

p <- ggboxplot(rt, x = "age", y = "TTC13",

color = "age", palette = "jco",

add = "jitter")+ylab("The expression of TTC13")

p + stat_compare_means(label.x = 1.5, label.y = 6)

#gender

compare_means(TTC13 ~ gender, data = rt, method = "anova")

p <- ggboxplot(rt, x = "gender", y = "TTC13",

color = "gender", palette = "jco",

add = "jitter")+ylab("The expression of TTC13")

p + stat_compare_means(label.x = 1.5, label.y = 6)

#### GSEA software version data preparation ####

load(file = "data/TCGA_KIRC_mRNA.Rdata")

gene <- "TTC13"

med=median(as.numeric(expr_TCGA_TPM_01A[gene,])) # Take the median of TTC13 expression

conditions=data.frame(sample=colnames(expr_TCGA_TPM_01A),

group=factor(ifelse(expr_TCGA_TPM_01A[gene,]>med,"high","low"),levels = c("low","high"))) %>%

column_to_rownames("sample")

high <- rownames(conditions)[conditions$group == "high"] #266

low <- rownames(conditions)[conditions$group == "low"] #267

expr_TCGA_TPM_01A <- expr_TCGA_TPM_01A[,c(high,low)]

### Board desktop version

### Docking Desktop Version from R Language

### Three files required

### Expression level gct, phenotype cls, gene set gmt

exprSet = expr_TCGA_TPM_01A

exprSet <- cbind(gene_id = rownames(exprSet), description = "na", exprSet)

## Create gct file

gct_file = paste0("my.gct")

sink(gct_file)

cat("#1.2\n")

cat(paste0(nrow(exprSet), "\t", (length(exprSet) - 2), "\n"))

sink()

write.table(exprSet, gct_file, append = T, quote = F, row.names = F, col.names = T,

sep = "\t")

# Create cls file

group_list <- c(rep("high",266),rep("low",267))

cls_file = paste0("my.cls")

sink(cls_file)

cat(paste0(length(group_list), "\t", length(unique(group_list)), "\t1\n"))

cat(paste0("#", paste(unique(group_list), collapse = "\t"), "\n"))

cat(paste(group_list, collapse = "\t"), "\n")

sink()

#### Single/Multiple Factor Analysis ####

load(file = "data/TTC13_clinic.Rdata")

rt <- rt %>%

select(c("time","status",

"age","gender","Stage","T_Stage","N_Stage","M_Stage","TTC13"))

# Single factor Cox regression

# Transforming factors into numbers

rt$T_Stage <- if_else(rt$T_Stage =="T4",4,

if_else(rt$T_Stage =="T3",3,

if_else(rt$T_Stage =="T2",2,1)))

rt$gender <- if_else(rt$gender == "female",0,1)

rt$Stage <- if_else(rt$Stage == "Stage IV",4,

if_else(rt$Stage == "Stage III",3,

if_else(rt$Stage == "Stage II",2,1)))

rt$N_Stage <- if_else(rt$N_Stage == "N1",2,1)

rt$M_Stage <- if_else(rt$M_Stage == "M0",0,1)

# Assuming we need to perform a univariate cox regression analysis on the following 5 features

covariates <- c("gender","age","Stage","T_Stage","N_Stage","M_Stage","TTC13")

# Construct survival analysis formulas for each variable separately

univ_formulas <-

sapply(covariates,

function(x)

as.formula(paste('Surv(time, status)~', x))) # Pay attention to changing the name of time and OS

# Loop through Cox regression analysis for each feature

univ_models <- lapply( univ_formulas, function(x){coxph(x, data = rt)}) # Pay attention to changing data

univ_results <- lapply(univ_models,

function(x){

x <- summary(x)

# Obtain p-value

p.value<-signif(x$wald["pvalue"], digits=4)

# Obtain HR

HR <-signif(x$coef[2], digits=4);

# Obtain 95% confidence interval

HR.confint.lower <- signif(x$conf.int[,"lower .95"], 4)

HR.confint.upper <- signif(x$conf.int[,"upper .95"],4)

lower <- HR.confint.lower

upper <- HR.confint.upper

HR <- HR

res<-c(p.value,HR,lower,upper)

names(res)<-c("pvalue","HR","lower","upper")

return(res)

})

# Convert to a data frame and transpose it

res <- t(as.data.frame(univ_results, check.names = FALSE))

as.data.frame(res)

write.table(file="output/TTC13_univariate_cox_result.txt",as.data.frame(res),quote=F,sep="\t")

library(forestploter)

# Read single factor results

dt <- data.table::fread("output/ TTC13_univariate_cox_result.txt",data.table = F)

dt$` ` <- paste(rep(" ", 20), collapse = " ")

dt$HR = round(dt$HR,4)

dt$pvalue = round(dt$pvalue,4)

dt$lower = round(dt$lower,4)

dt$upper = round(dt$upper,4)

dt$`HR (95% CI)` <- ifelse(is.na(dt$HR), "",

sprintf("%.3f (%.3f to %.3f)",

dt$HR, dt$lower, dt$upper))

tm <- forest_theme(base_size = 10,

# Confidence interval point shape, line type/color/width

ci_pch = 16, # point shape

ci_col = "red",

ci_lty = 1, # Line type

ci_lwd = 1.5, # line width

ci_Theight = 0.4, # Set T-tail at the end of CI

# Reference Line Weight/Type/Color

refline_lwd = 1,

refline_lty = "dashed",

refline_col = "grey20",

# Vertical line width/type/color

vertline_lwd = 1,

vertline_lty = "dashed",

vertline_col = "grey20",

# Change the summary color of padding and borders

summary_fill = "red",

summary_col = "red",

# Footnote font size/font style/color

footnote_cex = 0.6,

footnote_fontface = "italic",

footnote_col = "red")

plot <- forest(dt[, c(1,2,6,7)],

est = dt$HR,

lower = dt$lower,

upper = dt$upper,

ci_column = 3,theme = tm,ticks_at = c(0, 1, 5, 10,15,20,25,30),

ref_line = 1)# Default is 1

plot

# Multivariate Cox Regression Analysis of TCGA #

res.cox <- coxph(Surv(time, status) ~ age + Stage + T_Stage +

N_Stage + M_Stage + TTC13, data = rt)

x <- summary(res.cox)

pvalue=signif(as.matrix(x$coefficients)[,5],4)

HR=signif(as.matrix(x$coefficients)[,2],4)

low=signif(x$conf.int[,3],4)

high=signif(x$conf.int[,4],4)

multi_res=data.frame(pvalue=pvalue,

HR=HR,

lower=low,

upper=high,

stringsAsFactors = F

)

multi_res

write.table(file="output/TTC13multivariate_cox_result.txt",multi_res,quote=F,sep="\t")

## draw designs

library(forestploter)

# Read multi factor results

dt <- data.table::fread("output/TTC13multivariate_cox_result.txt",data.table = F)

dt$` ` <- paste(rep(" ", 20), collapse = " ")

dt$HR = round(dt$HR,4)

dt$pvalue = round(dt$pvalue,4)

dt$lower = round(dt$lower,4)

dt$upper = round(dt$upper,4)

dt$`HR (95% CI)` <- ifelse(is.na(dt$HR), "",

sprintf("%.3f (%.3f to %.3f)",

dt$HR, dt$lower, dt$upper))

tm <- forest_theme(base_size = 10,

# Confidence interval point shape, line type/color/width

ci_pch = 16, # point shape

ci_col = "red",

ci_lty = 1, # Line type

ci_lwd = 1.5, # line width

ci_Theight = 0.4, # Set T-tail at the end of CI

# Reference Line Weight/Type/Color

refline_lwd = 1,

refline_lty = "dashed",

refline_col = "grey20",

# Vertical line width/type/color

vertline_lwd = 1,

vertline_lty = "dashed",

vertline_col = "grey20",

# Change the summary color of padding and borders

summary_fill = "red",

summary_col = "red",

# Footnote font size/font style/color

footnote_cex = 0.6,

footnote_fontface = "italic",

footnote_col = "red")

plot <- forest(dt[, c(1,2,6,7)],

est = dt$HR,

lower = dt$lower,

upper = dt$upper,

ci_column = 3,theme = tm,ticks_at = c(0, 1, 5, 10,15,20,25,30),

ref_line = 1)# Default is 1

plot

####nomcox####

library(survival)

library(regplot)

library(rms)

load(file = "data/TTC13_clinic.Rdata")

rt <- rt %>%

select(c("time","status",

"age","gender","Stage","T_Stage","N_Stage","M_Stage","TTC13"))

nomcox=coxph(Surv(time, status) ~ . , data = rt)

regplot(nomcox,

plots = c("bars", "boxes"),

clickable=F,

title=NULL,

points=T,

droplines=T,

observation=rt[20,],

rank="sd",

failtime = c(1,3,5),

prfail = F)

# calibration curve

pdf("calibration.pdf",10,8)

#1 year

mx1 <- cph(Surv(time, status) ~TTC13, x=T, y=T, surv=T, data=rt, time.inc=1)

cal1 <- calibrate(mx1, cmethod="KM", method="boot", u=1, m=(nrow(rt)/3), B=1000)

plot(cal1, xlim=c(0,1), ylim=c(0,1),

xlab="Nomogram-predicted Overall survival (%)", ylab="Observed Overall survival (%)", lwd=1.5, col="green", sub=F)

#3 year

mx2 <- cph(Surv(time, status) ~ TTC13, x=T, y=T, surv=T, data=rt, time.inc=3)

cal2 <- calibrate(mx2, cmethod="KM", method="boot", u=2, m=(nrow(rt)/3), B=1000)

plot(cal2, xlim=c(0,1), ylim=c(0,1), xlab="", ylab="", lwd=1.5, col="blue", sub=F, add=T)

#5 year

mx3 <- cph(Surv(time, status) ~TTC13, x=T, y=T, surv=T, data=rt, time.inc=5)

cal3 <- calibrate(mx3, cmethod="KM", method="boot", u=3, m=(nrow(rt)/3), B=1000)

plot(cal3, xlim=c(0,1), ylim=c(0,1), xlab="", ylab="", lwd=1.5, col="red", sub=F, add=T)

legend('bottomright', c('1-year', '3-year', '5-year'),

col=c("green","blue","red"), lwd=1.5, bty = 'n')

dev.off()

#### Immune infiltration ####

library(e1071)

library(parallel)

library(preprocessCore)

source("CIBERSORT.R") # This side needs to read the ready-made R code in the folder

load(file = "data/TCGA_KIRC_mRNA.Rdata")

write.table(expr_TCGA_TPM_01A, file = "output/KIRC_mRNA_01A.txt",sep = "\t",row.names = T,col.names = NA,quote = F)

sig_matrix <- "LM22.txt" # Annotation file for immune cells

mixture_file = 'output/KIRC_mRNA_01A.txt' # Expression profile of tumor patients

res_cibersort <- CIBERSORT(sig_matrix, mixture_file, perm=100, QN=TRUE) # The running time here is longer, and 100 is the number of calculations. The larger the number, the more accurate the data

save(res_cibersort,file = "output/res_cibersort.Rdata") # Save intermediate files

library(limma)

library(reshape2)

library(ggpubr)

# Load cibersort data

load("output/res_cibersort.Rdata")

res_cibersort <- res_cibersort[,1:22]

data <- res_cibersort[,colSums(res_cibersort) > 0]

data <- as.data.frame(data)

risk <- t(expr_TCGA_TPM_01A)

sameSample=intersect(row.names(data), row.names(risk))

data=cbind(risk[sameSample,"TTC13",drop=F],data[sameSample,,drop=F])

corResult=data.frame()

for(i in colnames(data)){

tdc <- cor.test(data[,"TTC13"], data[,i], method = "pearson")

pvalue=tdc$p.value

corResult=rbind(corResult,

cbind(gene=i,

cor= tdc$estimate,

Low95CI=tdc$conf.int[1],

High95CI=tdc$conf.int[2],

pvalue=tdc$p.value)

)

}

outTab=corResult[as.numeric(corResult$pvalue)<0.05,]

#Plasma cells#

library(ggpubr)

ggscatter(data, x = "TTC13", y = "Plasma cells",

color = "black", size =2, # Points color and size

add = "reg.line", # Add regression line

add.params = list(color = "blue", fill = "gray"), # Customize regression line

conf.int = TRUE, # Add confidence interval

cor.coef = TRUE, # Add correlation coefficient. see ?stat_cor

cor.coeff.args = list(method = "pearson"))

#T cells CD4 naive#

library(ggpubr)

ggscatter(data, x = "TTC13", y = "T cells CD4 naive",

color = "black", size =2, # Points color and size

add = "reg.line", # Add regression line

add.params = list(color = "blue", fill = "gray"), # Customize regression line

conf.int = TRUE, # Add confidence interval

cor.coef = TRUE, # Add correlation coefficient. see ?stat_cor

cor.coeff.args = list(method = "pearson"))

#T cells CD4 memory resting#

library(ggpubr)

ggscatter(data, x = "TTC13", y = "T cells CD4 memory resting",

color = "black", size =2, # Points color and size

add = "reg.line", # Add regression line

add.params = list(color = "blue", fill = "gray"), # Customize regression line

conf.int = TRUE, # Add confidence interval

cor.coef = TRUE, # Add correlation coefficient. see ?stat_cor

cor.coeff.args = list(method = "pearson"))

#T cells follicular helper#

library(ggpubr)

ggscatter(data, x = "TTC13", y = "T cells follicular helper",

color = "black", size =2, # Points color and size

add = "reg.line", # Add regression line

add.params = list(color = "blue", fill = "gray"), # Customize regression line

conf.int = TRUE, # Add confidence interval

cor.coef = TRUE, # Add correlation coefficient. see ?stat_cor

cor.coeff.args = list(method = "pearson"))

#NK cells activated#

library(ggpubr)

ggscatter(data, x = "TTC13", y = "NK cells activated",

color = "black", size =2, # Points color and size

add = "reg.line", # Add regression line

add.params = list(color = "blue", fill = "gray"), # Customize regression line

conf.int = TRUE, # Add confidence interval

cor.coef = TRUE, # Add correlation coefficient. see ?stat_cor

cor.coeff.args = list(method = "pearson"))

#Macrophages M0#

library(ggpubr)

ggscatter(data, x = "TTC13", y = "Macrophages M0",

color = "black", size =2, # Points color and size

add = "reg.line", # Add regression line

add.params = list(color = "blue", fill = "gray"), # Customize regression line

conf.int = TRUE, # Add confidence interval

cor.coef = TRUE, # Add correlation coefficient. see ?stat_cor

cor.coeff.args = list(method = "pearson"))

#Macrophages M1#

library(ggpubr)

ggscatter(data, x = "TTC13", y = "Macrophages M1",

color = "black", size =2, # Points color and size

add = "reg.line", # Add regression line

add.params = list(color = "blue", fill = "gray"), # Customize regression line

conf.int = TRUE, # Add confidence interval

cor.coef = TRUE, # Add correlation coefficient. see ?stat_cor

cor.coeff.args = list(method = "pearson"))

#Dendritic cells activated#

library(ggpubr)

ggscatter(data, x = "TTC13", y = "Dendritic cells activated",

color = "black", size =2, # Points color and size

add = "reg.line", # Add regression line

add.params = list(color = "blue", fill = "gray"), # Customize regression line

conf.int = TRUE, # Add confidence interval

cor.coef = TRUE, # Add correlation coefficient. see ?stat_cor

cor.coeff.args = list(method = "pearson"))

#Mast cells resting#

library(ggpubr)

ggscatter(data, x = "TTC13", y = "Mast cells resting",

color = "black", size =2, # Points color and size

add = "reg.line", # Add regression line

add.params = list(color = "blue", fill = "gray"), # Customize regression line

conf.int = TRUE, # Add confidence interval

cor.coef = TRUE, # Add correlation coefficient. see ?stat_cor

cor.coeff.args = list(method = "pearson"))

#Eosinophils#

library(ggpubr)

ggscatter(data, x = "TTC13", y = "Eosinophils",

color = "black", size =2, # Points color and size

add = "reg.line", # Add regression line

add.params = list(color = "blue", fill = "gray"), # Customize regression line

conf.int = TRUE, # Add confidence interval

cor.coef = TRUE, # Add correlation coefficient. see ?stat_cor

cor.coeff.args = list(method = "pearson"))

####TIDE####

options(stringsAsFactors = FALSE) # Prohibit Chr from being converted to factor

# Expression Matrix

load(file = "data/TCGA_KIRC_mRNA.Rdata")

dat <- expr_TCGA_TPM_01A

TCGA_id <- colnames(expr_TCGA_TPM_01A)

med=median(as.numeric(expr_TCGA_TPM_01A["TTC13",]))

expr_TCGA_TPM_01A <- t(expr_TCGA_TPM_01A)

expr_TCGA_TPM_01A <- as.data.frame(expr_TCGA_TPM_01A)

sample <- ifelse(expr_TCGA_TPM_01A$TTC13 > med, "TTC13_high","TTC13_low")

sample <- factor(sample,levels = c("TTC13_high","TTC13_low")) # The purpose of factor is to rank normal first

metadata <- data.frame(TCGA_id,sample)

rownames(metadata) <- metadata$TCGA_id

# Clinical data

sameSample=intersect(colnames(dat),rownames(metadata))

dat=dat[,sameSample]

metadata=metadata[sameSample,]

ann <- metadata$sample

ann <- as.data.frame(ann)

rownames(ann) <- rownames(metadata)

colnames(ann)[1] <- "ImmClust"

TIDE <- dat

# In order to obtain better results, two direction media centers are used here

TIDE <- sweep(TIDE,2, apply(TIDE,2,median,na.rm=T))

TIDE <- sweep(TIDE,1, apply(TIDE,1,median,na.rm=T))

write.table(TIDE,"data/TIDE_input.self_subtract",sep = "\t",row.names = T,col.names = NA,quote = F)

#------------------------------------#

# Please refer to the TIDE tutorial. docx in the folder to complete this section #

TIDE.res <- read.csv("TIDE_output.csv",header = T,row.names = 1,check.names = F,stringsAsFactors = F)

TIDE.res <- TIDE.res[rownames(metadata),]

data=cbind(TIDE.res, metadata)

#TIDE#

library("RColorBrewer")

compare_means(TIDE ~ sample, data = data) # Gene, sample, data

p <- ggplot(data, aes(x = sample, y = TIDE, fill = sample)) +

geom_violin(trim = F) + scale_fill_brewer(palette = "Dark2") +

theme_classic() + geom_boxplot(width = 0.1, fill = "white") +

theme(text = element_text(size = 18),

axis.text.x = element_text(size = 18),

plot.title = element_text(hjust = 0.5),

legend.position = c(0.15,0.9)) +

xlab("sample") + ylab("TIDE")

p + stat_compare_means(label.x = 1.5, label.y = 6)

#TIDE#

library("RColorBrewer")

compare_means(TIDE ~ sample, data = data) # Gene, sample, data

p <- ggplot(data, aes(x = sample, y = TIDE, fill = sample)) +

geom_violin(trim = F) + scale_fill_brewer(palette = "Dark2") +

theme_classic() + geom_boxplot(width = 0.1, fill = "white") +

theme(text = element_text(size = 18),

axis.text.x = element_text(size = 18),

plot.title = element_text(hjust = 0.5),

legend.position = c(0.15,0.9)) +

xlab("sample") + ylab("TIDE")

p + stat_compare_means(label.x = 1.5, label.y = 6)

#MDSC#

library("RColorBrewer")

compare_means(MDSC ~ sample, data = data) # Gene, sample, data

p <- ggplot(data, aes(x = sample, y = MDSC, fill = sample)) +

geom_violin(trim = F) + scale_fill_brewer(palette = "Dark2") +

theme_classic() + geom_boxplot(width = 0.1, fill = "white") +

theme(text = element_text(size = 18),

axis.text.x = element_text(size = 18),

plot.title = element_text(hjust = 0.5),

legend.position = c(0.15,0.9)) +

xlab("sample") + ylab("MDSC")

p + stat_compare_means(label.x = 1.5, label.y = 0.8)

#Exclusion#

library("RColorBrewer")

compare_means(Exclusion ~ sample, data = data) # Gene, sample, data

p <- ggplot(data, aes(x = sample, y = Exclusion, fill = sample)) +

geom_violin(trim = F) + scale_fill_brewer(palette = "Dark2") +

theme_classic() + geom_boxplot(width = 0.1, fill = "white") +

theme(text = element_text(size = 18),

axis.text.x = element_text(size = 18),

plot.title = element_text(hjust = 0.5),

legend.position = c(0.15,0.9)) +

xlab("sample") + ylab("Exclusion")

p + stat_compare_means(label.x = 1.5, label.y = 0.8)

#CD8#

library("RColorBrewer")

compare_means(CD8 ~ sample, data = data) # Gene, sample, data

p <- ggplot(data, aes(x = sample, y = CD8, fill = sample)) +

geom_violin(trim = F) + scale_fill_brewer(palette = "Dark2") +

theme_classic() + geom_boxplot(width = 0.1, fill = "white") +

theme(text = element_text(size = 18),

axis.text.x = element_text(size = 18),

plot.title = element_text(hjust = 0.5),

legend.position = c(0.15,0.9)) +

xlab("sample") + ylab("CD8")

p + stat_compare_means(label.x = 1.5, label.y = 0.8)

#Merck18#

library("RColorBrewer")

compare_means(Merck18 ~ sample, data = data) # Gene, sample, data

p <- ggplot(data, aes(x = sample, y = Merck18, fill = sample)) +

geom_violin(trim = F) + scale_fill_brewer(palette = "Dark2") +

theme_classic() + geom_boxplot(width = 0.1, fill = "white") +

theme(text = element_text(size = 18),

axis.text.x = element_text(size = 18),

plot.title = element_text(hjust = 0.5),

legend.position = c(0.15,0.9)) +

xlab("sample") + ylab("Merck18")

p + stat_compare_means(label.x = 1.5, label.y = 0.25)

#TAM M2#

library("RColorBrewer")

colnames(data)[14] <- "TAMM2"

compare_means(TAMM2 ~ sample, data = data) # Gene, sample, data

p <- ggplot(data, aes(x = sample, y = TAMM2, fill = sample)) +

geom_violin(trim = F) + scale_fill_brewer(palette = "Dark2") +

theme_classic() + geom_boxplot(width = 0.1, fill = "white") +

theme(text = element_text(size = 18),

axis.text.x = element_text(size = 18),

plot.title = element_text(hjust = 0.5),

legend.position = c(0.15,0.9)) +

xlab("sample") + ylab("TAM M2")

p + stat_compare_means(label.x = 1.5, label.y = 0.25)

#CAF#

library("RColorBrewer")

compare_means(CAF ~ sample, data = data) # Gene, sample, data

p <- ggplot(data, aes(x = sample, y = CAF, fill = sample)) +

geom_violin(trim = F) + scale_fill_brewer(palette = "Dark2") +

theme_classic() + geom_boxplot(width = 0.1, fill = "white") +

theme(text = element_text(size = 18),

axis.text.x = element_text(size = 18),

plot.title = element_text(hjust = 0.5),

legend.position = c(0.15,0.9)) +

xlab("sample") + ylab("CAF")

p + stat_compare_means(label.x = 1.5, label.y = 0.8)

#Dysfunction#

library("RColorBrewer")

compare_means(Dysfunction ~ sample, data = data) # Gene, sample, data

p <- ggplot(data, aes(x = sample, y = Dysfunction, fill = sample)) +

geom_violin(trim = F) + scale_fill_brewer(palette = "Dark2") +

theme_classic() + geom_boxplot(width = 0.1, fill = "white") +

theme(text = element_text(size = 18),

axis.text.x = element_text(size = 18),

plot.title = element_text(hjust = 0.5),

legend.position = c(0.15,0.9)) +

xlab("sample") + ylab("Dysfunction")

p + stat_compare_means(label.x = 1.5, label.y = 4)

#CD274#

library("RColorBrewer")

compare_means(CD274 ~ sample, data = data) # Gene, sample, data

p <- ggplot(data, aes(x = sample, y = CD274, fill = sample)) +

geom_violin(trim = F) + scale_fill_brewer(palette = "Dark2") +

theme_classic() + geom_boxplot(width = 0.1, fill = "white") +

theme(text = element_text(size = 18),

axis.text.x = element_text(size = 18),

plot.title = element_text(hjust = 0.5),

legend.position = c(0.15,0.9)) +

xlab("sample") + ylab("CD274")

p + stat_compare_means(label.x = 1.5, label.y = 4)

#MSI#

library("RColorBrewer")

colnames(data)[5] <- "MSI"

compare_means(MSI ~ sample, data = data) # Gene, sample, data

p <- ggplot(data, aes(x = sample, y = MSI, fill = sample)) +

geom_violin(trim = F) + scale_fill_brewer(palette = "Dark2") +

theme_classic() + geom_boxplot(width = 0.1, fill = "white") +

theme(text = element_text(size = 18),

axis.text.x = element_text(size = 18),

plot.title = element_text(hjust = 0.5),

legend.position = c(0.15,0.9)) +

xlab("sample") + ylab("MSI")

p + stat_compare_means(label.x = 1.5, label.y = 1.5)

#IFNG#

library("RColorBrewer")

compare_means(IFNG ~ sample, data = data) # Gene, sample, data

p <- ggplot(data, aes(x = sample, y = IFNG, fill = sample)) +

geom_violin(trim = F) + scale_fill_brewer(palette = "Dark2") +

theme_classic() + geom_boxplot(width = 0.1, fill = "white") +

theme(text = element_text(size = 18),

axis.text.x = element_text(size = 18),

plot.title = element_text(hjust = 0.5),

legend.position = c(0.15,0.9)) +

xlab("sample") + ylab("IFNG")

p + stat_compare_means(label.x = 1.5, label.y = 4)

#### Prediction of drug sensitivity oncoptosis ####

#TCGA#

source("CALCPHENOTYPE.R")

library(sva)

library(preprocessCore)

library(ridge)

library(glmnet)

library(car)

library(tidyverse)

library(impute)

library(ggplot2)

library(cowplot)

Sys.setenv(LANGUAGE = "en") # Display English error messages

options(stringsAsFactors = FALSE) # Prohibit Chr from being converted to factor

# Expression Matrix

load(file = "data/TCGA_KIRC_mRNA.Rdata")

dat <- expr_TCGA_TPM_01A

# Clinical data

TCGA_id <- colnames(expr_TCGA_TPM_01A)

med=median(as.numeric(expr_TCGA_TPM_01A["TTC13",]))

expr_TCGA_TPM_01A <- t(expr_TCGA_TPM_01A)

expr_TCGA_TPM_01A <- as.data.frame(expr_TCGA_TPM_01A)

sample <- ifelse(expr_TCGA_TPM_01A$TTC13 > med, "TTC13_high","TTC13_low")

sample <- factor(sample,levels = c("TTC13_high","TTC13_low")) # The purpose of factor is to rank normal first

metadata <- data.frame(TCGA_id,sample)

rownames(metadata) <- metadata$TCGA_id

sameSample=intersect(colnames(dat),rownames(metadata))

dat=dat[,sameSample]

rt=metadata[sameSample,]

ann <- rt$sample

ann <- as.data.frame(ann)

rownames(ann) <- rownames(rt)

colnames(ann)[1] <- "ImmClust"

table(ann$ImmClust)

# Building Test Set Data

testingExprData <- as.matrix(dat[,rownames(ann)])

# Preparation Data 1: Cell Line Expression

trainingExprData <- readRDS(file='GDSC2_Expr_short.rds') # The data type is matrix, and the cell line expression is a behavioral gene. It is listed as a cell line, and the input value is a matrix of the expression profile. The expression profile needs to be log2 standardized

# Preparation Data 2: Cell Line Corresponding Drug Sensitivity

trainingPtype <- readRDS(file = "GDSC2_Res.rds") # The data type is matrix. Cell line drug sensitivity is a behavioral cell line, listed as a drug, and the input value is a matrix of drug sensitivity. The data generally contains NA and needs to be filtered or filled in

trainingPtype <- trainingPtype[,apply(trainingPtype, 2, function(x) {sum(is.na(x)) < 0.2 * nrow(trainingPtype)})] # Remove drugs that are missing in over 20% of the samples

trainingPtype <- trainingPtype[apply(trainingPtype, 1, function(x) {sum(is.na(x)) < 0.2 * ncol(trainingPtype)}),] # Remove samples that are missing in over 20% of the medication

trainingPtype <- t(impute.knn(t(trainingPtype))$data) # KNN filling missing values

trainingPtype <- exp(trainingPtype) # Exponentiation is done here based on the characteristics of the data, as the original data is logarithmic, but CALCPHENOTYPE. R will perform a power transformation in one step to avoid duplication. Here, the data size is restored

batchCorrect <- "eb" # But my test set is standardized RNA seq, which is similar to the chip signal strength, so I still choose eb

# Whether to perform power transformation option: default

powerTransformPhenotype <- TRUE

# Low mutation gene filtering threshold option: default to 0.2

removeLowVaryingGenes <- 0.2

# Data type options used when filtering genes: 'homogenizeData' (data after batch effect elimination) or 'rawData' (raw data)

removeLowVaringGenesFrom <- "homogenizeData"

# Determine the minimum number of training samples (this problem generally does not exist because GDSC, CTRP, and PRISM all have many cell lines)

minNumSamples <- 10

# Determine how to handle duplicate gene options: -1 is to ask the user, 1 is to take the mean, and 2 is to remove duplicates

selection <- 1

# Determine whether to print the results option: default

printOutput <- FALSE # I won't do it here to avoid the final markdown file being too long. When I run it myself, I can change it to TRUE to see the progress

# Determine whether to use PCA for dimensionality reduction of gene expression option: default not to perform

# Note that if the option 'report' is selected_ If pca=TRUE, the above options must be true

pcr <- FALSE

report_pc <- FALSE

# Determine whether it is necessary to list correlation results to determine potential drug biomarkers (personally, I don't think it is necessary. The search for drug targets can refer to Figure Ya212drugTarget, which is too "simple and rough")

cc <- FALSE

# Determine whether to input the goodness of fit value R ^ 2 of the model (the degree of agreement between the model backpropagation of the cell line training set and the prediction results of the training set)

rsq <- FALSE # If you want to abuse yourself and check the accuracy of the model, you can change it to TRUE

# Determine the explanatory power that principal component analysis needs to achieve when pcr=TRUE, with a default of 80, which means that the total explanatory power of the obtained principal components should be greater than 80%

percent <- 80

# Build a model and predict that this function will generate calcPhenotype in the current directory_ Output and input the drug sensitivity prediction results into this directory, named DrugPredications.csv calcPhenotype (trainingExprData=as. matrix (trainingExprData). The # function will match the shared samples of cell line expression and drug sensitivity on its own

trainingPtype = trainingPtype, # But if you want to choose a special cell line type, such as the "reproductive system", "digestive system", etc., you need to pre screen the cell line in advance, and the function does not provide this operation

testExprData = as.matrix(testingExprData),

batchCorrect = batchCorrect,

powerTransformPhenotype = powerTransformPhenotype,

removeLowVaryingGenes = removeLowVaryingGenes,

minNumSamples = minNumSamples,

selection = selection,

printOutput = printOutput,

pcr = pcr,

removeLowVaringGenesFrom = removeLowVaringGenesFrom,

report_pc = report_pc,

cc = cc,

percent = percent,

rsq = rsq)

drugsen <- read.csv("./calcPhenotype_Output/DrugPredictions.csv",row.names = 1,check.names = F,stringsAsFactors = F,header = T)

outTab <- NULL

for (drug in colnames(drugsen)) {

tmp <- data.frame(sen = drugsen[,drug],

group = ann[rownames(drugsen),"ImmClust"],

row.names = rownames(drugsen),

stringsAsFactors = F)

avg <- as.numeric(by(tmp$sen, tmp$group, mean))

wt <- wilcox.test(sen~group,data = tmp)

outTab <- rbind.data.frame(outTab,

data.frame(drug = drug,

avg1 = avg[1],

avg2 = avg[2],

p = wt$p.value,

stringsAsFactors = F),

stringsAsFactors = F)

}

outTab$fc <- outTab$avg2/outTab$avg1

outTab$log2fc <- log2(outTab$fc)

# Output statistical results to a file

write.table(outTab,"output_wilcox test for potential drugs.txt",sep = "\t",row.names = F,col.names = T,quote = F)

# COLOR

jco <- c("#EABF00", "#2874C5")

plotp <- list()

GCP.drug <- outTab[which(outTab$p < 0.05 & outTab$fc > 0),"drug"] # This is the threshold I randomly selected to control the number of drugs

for (drug in GCP.drug) {

predictedBoxdat <- data.frame("response" = drugsen[rownames(ann),drug],

"Group" = ann$ImmClust,

row.names = rownames(ann))

# draw

my_comparisons <- list(c("TTC13_high", "TTC13_low"))

p <- ggplot(data = predictedBoxdat, aes(x=Group, y=response))

p <- p + geom_boxplot(aes(fill = Group)) +

scale_fill_manual(values = jco[1:length(unique(predictedBoxdat$Group))]) + # Customize the color scheme of the box

theme_classic() +

theme(legend.position="none") + # Italic font

theme(axis.text.x = element_text(angle = 45, hjust = 1,size = 12), plot.title = element_text(size = 12, hjust = 0.5)) +

xlab("") + ylab("Drug sensitivity") + # This may be AUC or IC50, depending on the specific situation

ggtitle(drug)+stat_compare_means(comparisons = my_comparisons, method = "t.test") # Add title

# Draw the results of each drug in a separate file

ggsave(paste("boxplot of", drug, "sensitivity.pdf"), width = 4, height = 5)

plotp[[drug]] <- p # Save in list for merging images

}

# Merge Pictures

p <- plot_grid(plotlist = plotp,nrow = 2) # The title can be AI pulled down to the appropriate location, as shown in the example text

p

ggsave("boxplot of predicted drug sensitivity.pdf", width = 6, height = 5)

#### Pancancer analysis ####

# Single gene pan cancer expression matrix #

library(limma)

gene="TTC13"

files=dir()

files=grep("^symbol.", files, value=T)

geneList=list()

for(i in files){

CancerType=gsub("symbol\\.|\\.txt", "", i)

rt=read.table(i, header=T, sep="\t", check.names=F)

geneList[[CancerType]]=as.vector(rt[,1])

}

interGenes=Reduce(intersect, geneList)

outTab=data.frame()

allTab=data.frame()

j=0

for(i in files){

CancerType=gsub("symbol\\.|\\.txt", "", i)

rt=read.table(i, header=T, sep="\t", check.names=F)

rt=as.matrix(rt)

rownames(rt)=rt[,1]

exp=rt[,2:ncol(rt)]

dimnames=list(rownames(exp), colnames(exp))

data=matrix(as.numeric(as.matrix(exp)), nrow=nrow(exp), dimnames=dimnames)

data=t(avereps(data))

row.names(data)=gsub(".$", "", row.names(data))

data=t(avereps(data))

group=sapply(strsplit(colnames(data),"\\-"), "[", 4)

group=sapply(strsplit(group,""), "[", 1)

Type=ifelse(group==0, "Tumor", "Normal")

geneExp=t(data[gene,,drop=F])

outTab=rbind(outTab, cbind(geneExp,Type,CancerType))

j=j+1

if(j==1){

allTab=data[interGenes,group==0]

}else{

allTab=cbind(allTab, data[interGenes,group==0])

}

}

# Output single gene expression files

out=cbind(ID=row.names(outTab), outTab)

write.table(out, file="geneExp.txt", sep="\t", quote=F, row.names=F)

# Output expression files for all tumors

allTab=allTab[rowMeans(allTab)>0.1,]

allTab=cbind(ID=row.names(allTab), allTab)

write.table(allTab, file="merge.txt", sep="\t", quote=F, row.names=F)

#### TMB radar map ####

library(fmsb)

expFile="geneExp.txt"

tmbFile="TMB.txt"

col="red"

exp=read.table(expFile, header=T, sep="\t", check.names=F, row.names=1)

exp=exp[(exp[,"Type"]=="Tumor"),]

TMB=read.table(tmbFile, header=T, sep="\t", check.names=F, row.names=1)

TMB=as.matrix(TMB)

row.names(TMB)=gsub(".$", "", row.names(TMB))

sameSample=intersect(row.names(TMB), row.names(exp))

TMB=TMB[sameSample,]

exp=exp[sameSample,]

outTab=data.frame()

fmsbTab=data.frame()

for(i in levels(factor(exp[,"CancerType"]))){

exp1=exp[(exp[,"CancerType"]==i),]

TMB1=TMB[(TMB[,"CancerType"]==i),]

x=as.numeric(TMB1[,1])

y=as.numeric(exp1[,1])

corT=cor.test(x,y,method="spearman")

cor=corT$estimate

pValue=corT$p.value

sig=ifelse(pValue<0.001,"***",ifelse(pValue<0.01,"**",ifelse(pValue<0.05,"*"," ")))

outTab=rbind(outTab, cbind(CancerType=i,cor=cor,pValue=pValue))

fmsbTab=rbind(fmsbTab, cbind(CancerType=paste0(i,sig),cor=cor))

}

write.table(outTab,file="cor.result.txt",sep="\t",row.names=F,quote=F)

write.table(t(fmsbTab),file="fmsbInput.txt",sep="\t",col.names=F,quote=F)

data=read.table("fmsbInput.txt", header=T, sep="\t", check.names=F, row.names=1)

maxValue=ceiling(max(abs(data))*10)/10

data=rbind(rep(maxValue,ncol(data)),rep(-maxValue,ncol(data)),data)

pdf(file="radar.pdf", width=7, height=7)

radarchart(data, axistype=1,

title="Tumor mutation burden",

pcol=col,

plwd=2 ,

plty=1,

cglcol="grey",

cglty=1,

caxislabels=seq(-maxValue,maxValue,maxValue/2),

cglwd=1.2,

axislabcol="blue",

vlcex=0.8

)

dev.off()

#### MSI radar map ####

library(fmsb)

expFile="geneExp.txt"

msiFile="MSI.txt"

col="blue"

exp=read.table(expFile, header=T, sep="\t", check.names=F, row.names=1)

exp=exp[(exp[,"Type"]=="Tumor"),]

MSI=read.table(msiFile, header=T, sep="\t", check.names=F, row.names=1)

MSI=as.matrix(MSI)

row.names(MSI)=gsub(".$", "", row.names(MSI))

sameSample=intersect(row.names(MSI), row.names(exp))

MSI=MSI[sameSample,]

exp=exp[sameSample,]

outTab=data.frame()

fmsbTab=data.frame()

for(i in levels(factor(exp[,"CancerType"]))){

exp1=exp[(exp[,"CancerType"]==i),]

MSI1=MSI[(MSI[,"CancerType"]==i),]

x=as.numeric(MSI1[,1])

y=as.numeric(exp1[,1])

corT=cor.test(x,y,method="spearman")

cor=corT$estimate

pValue=corT$p.value

sig=ifelse(pValue<0.001,"***",ifelse(pValue<0.01,"**",ifelse(pValue<0.05,"*"," ")))

outTab=rbind(outTab, cbind(CancerType=i,cor=cor,pValue=pValue))

fmsbTab=rbind(fmsbTab, cbind(CancerType=paste0(i,sig),cor=cor))

}

write.table(outTab,file="cor.result.txt",sep="\t",row.names=F,quote=F)

write.table(t(fmsbTab),file="fmsbInput.txt",sep="\t",col.names=F,quote=F)

data=read.table("fmsbInput.txt", header=T, sep="\t", check.names=F, row.names=1)

maxValue=ceiling(max(abs(data))*10)/10

data=rbind(rep(maxValue,ncol(data)),rep(-maxValue,ncol(data)),data)

pdf(file="radar.pdf", width=7, height=7)

radarchart(data, axistype=1,

title="Microsatellite instability",

pcol=col,

plwd=2 ,

plty=1,

cglcol="grey",

cglty=1,

caxislabels=seq(-maxValue,maxValue,maxValue/2),

cglwd=1.2,

axislabcol="blue",

vlcex=0.8

)

dev.off()

#' CIBERSORT R script v1.03 (last updated 07-10-2015)

#' Note: Signature matrix construction is not currently available; use java version for full functionality.

#' Author: Aaron M. Newman, Stanford University (amnewman@stanford.edu)

#' Requirements:

#' R v3.0 or later. (dependencies below might not work properly with earlier versions)

#' install.packages('e1071')

#' install.pacakges('parallel')

#' install.packages('preprocessCore')

#' if preprocessCore is not available in the repositories you have selected, run the following:

#' source("http://bioconductor.org/biocLite.R")

#' biocLite("preprocessCore")

#' Windows users using the R GUI may need to Run as Administrator to install or update packages.

#' This script uses 3 parallel processes. Since Windows does not support forking, this script will run

#' single-threaded in Windows.

#'

#' Usage:

#' Navigate to directory containing R script

#'

#' In R:

#' source('CIBERSORT.R')

#' results <- CIBERSORT('sig_matrix_file.txt','mixture_file.txt', perm, QN)

#'

#' Options:

#' i) perm = No. permutations; set to >=100 to calculate p-values (default = 0)

#' ii) QN = Quantile normalization of input mixture (default = TRUE)

#'

#' Input: signature matrix and mixture file, formatted as specified at http://cibersort.stanford.edu/tutorial.php

#' Output: matrix object containing all results and tabular data written to disk 'CIBERSORT-Results.txt'

#' License: http://cibersort.stanford.edu/CIBERSORT_License.txt

#' Core algorithm

#' @param X cell-specific gene expression

#' @param y mixed expression per sample

#' @export

CoreAlg <- function(X, y){

#try different values of nu

svn_itor <- 3

res <- function(i){

if(i==1){nus <- 0.25}

if(i==2){nus <- 0.5}

if(i==3){nus <- 0.75}

model<-e1071::svm(X,y,type="nu-regression",kernel="linear",nu=nus,scale=F)

model

}

if(Sys.info()['sysname'] == 'Windows') out <- parallel::mclapply(1:svn_itor, res, mc.cores=1) else

out <- parallel::mclapply(1:svn_itor, res, mc.cores=svn_itor)

nusvm <- rep(0,svn_itor)

corrv <- rep(0,svn_itor)

#do cibersort

t <- 1

while(t <= svn_itor) {

weights = t(out[[t]]$coefs) %*% out[[t]]$SV

weights[which(weights<0)]<-0

w<-weights/sum(weights)

u <- sweep(X,MARGIN=2,w,'*')

k <- apply(u, 1, sum)

nusvm[t] <- sqrt((mean((k - y)^2)))

corrv[t] <- cor(k, y)

t <- t + 1

}

#pick best model

rmses <- nusvm

mn <- which.min(rmses)

model <- out[[mn]]

#get and normalize coefficients

q <- t(model$coefs) %*% model$SV

q[which(q<0)]<-0

w <- (q/sum(q))

mix_rmse <- rmses[mn]

mix_r <- corrv[mn]

newList <- list("w" = w, "mix_rmse" = mix_rmse, "mix_r" = mix_r)

}

#' do permutations

#' @param perm Number of permutations

#' @param X cell-specific gene expression

#' @param y mixed expression per sample

#' @export

doPerm <- function(perm, X, Y){

itor <- 1

Ylist <- as.list(data.matrix(Y))

dist <- matrix()

while(itor <= perm){

#print(itor)

#random mixture

yr <- as.numeric(Ylist[sample(length(Ylist),dim(X)[1])])

#standardize mixture

yr <- (yr - mean(yr)) / sd(yr)

#run CIBERSORT core algorithm

result <- CoreAlg(X, yr)

mix_r <- result$mix_r

#store correlation

if(itor == 1) {dist <- mix_r}

else {dist <- rbind(dist, mix_r)}

itor <- itor + 1

}

newList <- list("dist" = dist)

}

#' Main functions

#' @param sig_matrix file path to gene expression from isolated cells

#' @param mixture_file heterogenous mixed expression

#' @param perm Number of permutations

#' @param QN Perform quantile normalization or not (TRUE/FALSE)

#' @export

CIBERSORT <- function(sig_matrix, mixture_file, perm=0, QN=TRUE){

#read in data

X <- read.table(sig_matrix,header=T,sep="\t",row.names=1,check.names=F)

Y <- read.table(mixture_file, header=T, sep="\t", check.names=F)

Y <- Y[!duplicated(Y[,1]),]

rownames(Y)<-Y[,1]

Y<-Y[,-1]

X <- data.matrix(X)

Y <- data.matrix(Y)

#order

X <- X[order(rownames(X)),]

Y <- Y[order(rownames(Y)),]

P <- perm #number of permutations

#anti-log if max < 50 in mixture file

if(max(Y) < 50) {Y <- 2^Y}

#quantile normalization of mixture file

if(QN == TRUE){

tmpc <- colnames(Y)

tmpr <- rownames(Y)

Y <- preprocessCore::normalize.quantiles(Y)

colnames(Y) <- tmpc

rownames(Y) <- tmpr

}

#intersect genes

Xgns <- row.names(X)

Ygns <- row.names(Y)

YintX <- Ygns %in% Xgns

Y <- Y[YintX,]

XintY <- Xgns %in% row.names(Y)

X <- X[XintY,]

#standardize sig matrix

X <- (X - mean(X)) / sd(as.vector(X))

# Matrix X is an LM22 matrix

# Matrix Y is the matrix to be predicted

# And then

#empirical null distribution of correlation coefficients

if(P > 0) {nulldist <- sort(doPerm(P, X, Y)$dist)}

#print(nulldist)

header <- c('Mixture',colnames(X),"P-value","Correlation","RMSE")

#print(header)

output <- matrix()

itor <- 1

mixtures <- dim(Y)[2]

pval <- 9999

#iterate through mixtures

while(itor <= mixtures){

y <- Y[,itor]

#standardize mixture

y <- (y - mean(y)) / sd(y)

#run SVR core algorithm

result <- CoreAlg(X, y)

#get results

w <- result$w

mix_r <- result$mix_r

mix_rmse <- result$mix_rmse

#calculate p-value

if(P > 0) {pval <- 1 - (which.min(abs(nulldist - mix_r)) / length(nulldist))}

#print output

out <- c(colnames(Y)[itor],w,pval,mix_r,mix_rmse)

if(itor == 1) {output <- out}

else {output <- rbind(output, out)}

itor <- itor + 1

}

#save results

write.table(rbind(header,output), file="CIBERSORT-Results.txt", sep="\t", row.names=F, col.names=F, quote=F)

#return matrix object containing all results

obj <- rbind(header,output)

obj <- obj[,-1]

obj <- obj[-1,]

obj <- matrix(as.numeric(unlist(obj)),nrow=nrow(obj))

rownames(obj) <- colnames(Y)

colnames(obj) <- c(colnames(X),"P-value","Correlation","RMSE")

obj

}

#' This function performs variable selection on gene expression matrices.

#' It can, for instance, remove genes with low variation.

#' @param exprMat A matrix of gene expression levels. rownames() are genes, and colnames() are samples.

#' @param removeLowVaryingGenes The proportion of low varying genes to be removed.The default is .2

#' @return A vector of row/genes to keep.

#' @export

doVariableSelection <- function(exprMat, removeLowVaryingGenes=.2)

{

vars <- apply(exprMat, 1, var)

return(order(vars, decreasing=TRUE)[seq(1:as.integer(nrow(exprMat)*(1-removeLowVaryingGenes)))])

}

#'This function takes two gene expression matrices (like trainExprMat and testExprMat) and returns homogenized versions of the matrices by employing the homogenization method specified.

#'By default, the Combat method from the sva library is used.

#'In both matrices, genes are row names and samples are column names.

#'It will deal with duplicated gene names, as it subsets and orders the matrices correctly.

#'@param testExprMat A gene expression matrix for samples on which we wish to predict a phenotype.Genes are rows, samples are columns.

#'@param trainExprMat A gene expression matrix for samples for which the phenotype is already known.Genes are rows, samples are columns.

#'@param batchCorrect The type of batch correction to be used. Options are 'eb' for Combat, 'none', or 'qn' for quantile normalization.

#'#The default is 'eb'.

#'@param selection This parameter can be used to specify how duplicates are handled. The default value of -1 means to ask the user.

#'#Other options include '1' to summarize duplicates by their mean, and '2'to discard all duplicated genes.

#'@param printOutput To suppress output, set to false. Default is TRUE.

#'@import sva

#'@import preprocessCore

#'@keywords Homogenize gene expression data.

#'@return A list containing two entries $train and $test, which are the homogenized input matrices.

#'@export

homogenizeData<-function (testExprMat, trainExprMat, batchCorrect = "eb", selection = -1, printOutput = TRUE)

{

#Check the batchCorrect parameter

if (!(batchCorrect %in% c("eb", "qn", "none",

"rank", "rank_then_eb", "standardize")))

stop("\"batchCorrect\" must be one of \"eb\", \"qn\", \"rank\", \"rank_then_eb\", \"standardize\" or \"none\"")

#Check if both row and column names have been specified.

if (is.null(rownames(trainExprMat)) || is.null(rownames(testExprMat))) {

stop("ERROR: Gene identifiers must be specified as \"rownames()\" on both training and test expression matrices. Both matices must have the same type of gene identifiers.")

}

#Check that some of the row names overlap between both datasets (print an error if none overlap)

if (sum(rownames(trainExprMat) %in% rownames(testExprMat)) == 0) {

stop("ERROR: The rownames() of the supplied expression matrices do not match. Note that these are case-sensitive.")

}

else {

if (printOutput)

message(paste("\n", sum(rownames(trainExprMat) %in% rownames(testExprMat)), " gene identifiers overlap between the supplied expression matrices... \n", paste = ""))

}

#If there are duplicate gene names, give the option of removing them or summarizing them by their mean.

if ((sum(duplicated(rownames(trainExprMat))) > 0) || sum(sum(duplicated(rownames(testExprMat))) > 0)) {

if (selection == -1) {

message("\nExpression matrix contain duplicated gene identifiers (i.e. duplicate rownames()), how would you like to proceed:")

message("\n1. Summarize duplicated gene ids by their mean value (acceptable in most cases)")

message("\n2. Disguard all duplicated genes (recommended if unsure)")

message("\n3. Abort (if you want to deal with duplicate genes ids manually)\n")

}

while (is.na(selection) | selection <= 0 | selection > 3) {

selection <- readline("Selection: ")

selection <- ifelse(grepl("[^1-3.]", selection), -1, as.numeric(selection))

}

message("\n")

if (selection == 1) #Summarize duplicates by their mean.

{

if ((sum(duplicated(rownames(trainExprMat))) > 0)) {

trainExprMat <- summarizeGenesByMean(trainExprMat)

}

if ((sum(duplicated(rownames(testExprMat))) > 0)) {

testExprMat <- summarizeGenesByMean(testExprMat)

}

}

else if (selection == 2) #Disguard all duplicated genes.

{

if ((sum(duplicated(rownames(trainExprMat))) > 0)) {

keepGenes <- names(which(table(rownames(trainExprMat)) == 1))

trainExprMat <- trainExprMat[keepGenes, ]

}

if ((sum(duplicated(rownames(testExprMat))) > 0)) {

keepGenes <- names(which(table(rownames(testExprMat)) == 1))

testExprMat <- testExprMat[keepGenes, ]

}

}

else {

stop("Exectution Aborted!")

}

}

#Subset and order gene ids on the expression matrices.

commonGenesIds <- rownames(trainExprMat)[rownames(trainExprMat) %in%

rownames(testExprMat)]

trainExprMat <- trainExprMat[commonGenesIds, ]

testExprMat <- testExprMat[commonGenesIds, ]

#Subset and order the 2 expression matrices.

if (batchCorrect == "eb") {

#Subset to common genes and batch correct using ComBat.

dataMat <- cbind(trainExprMat, testExprMat)

mod <- data.frame(`(Intercept)` = rep(1, ncol(dataMat)))

rownames(mod) <- colnames(dataMat)

whichbatch <- as.factor(c(rep("train", ncol(trainExprMat)),

rep("test", ncol(testExprMat))))

# Added

# Filter out genes with low variances to make sure comBat run correctly

dataMat <- cbind(trainExprMat, testExprMat)

gene_vars = apply(dataMat, 1, var)

genes<-as.vector(gene_vars)

if (length(which(genes <= 1e-3) != 0)){ #If some genes have low variances (if the variance is not 0), remove them.

dataMat = dataMat[-(which(genes <= 1e-3)),]

}

# End added

combatout <- ComBat(dataMat, whichbatch, mod = mod)

return(list(train = combatout[, whichbatch == "train"],

test = combatout[, whichbatch == "test"], selection = selection))

}

else if (batchCorrect == "standardize") #Standardize to mean 0 and variance 1 in each dataset using a non EB based approach.

{

for (i in 1:nrow(trainExprMat)) {

row <- trainExprMat[i, ]

trainExprMat[i, ] <- ((row - mean(row))/sd(row))

}

for (i in 1:nrow(testExprMat)) {

row <- testExprMat[i, ]

testExprMat[i, ] <- ((row - mean(row))/sd(row))

}

return(list(train = trainExprMat, test = testExprMat,

selection = selection))

}

else if (batchCorrect == "rank") #The random-rank transform approach, that may be better when applying models to RNA-seq data.

{

for (i in 1:nrow(trainExprMat)) {

trainExprMat[i, ] <- rank(trainExprMat[i, ], ties.method = "random")

}

for (i in 1:nrow(testExprMat)) {

testExprMat[i, ] <- rank(testExprMat[i, ], ties.method = "random")

}

return(list(train = trainExprMat, test = testExprMat,

selection = selection))

}

else if (batchCorrect == "rank_then_eb") #Rank-transform the RNAseq data, then apply ComBat

{

#First, rank transform the RNA-seq data.

for (i in 1:nrow(testExprMat)) {

testExprMat[i, ] <- rank(testExprMat[i, ], ties.method = "random")

}

#Subset to common genes and batch correct using ComBat.

dataMat <- cbind(trainExprMat, testExprMat)

mod <- data.frame(`(Intercept)` = rep(1, ncol(dataMat)))

rownames(mod) <- colnames(dataMat)

whichbatch <- as.factor(c(rep("train", ncol(trainExprMat)),

rep("test", ncol(testExprMat))))

combatout <- ComBat(dataMat, whichbatch, mod = mod)

return(list(train = combatout[, whichbatch == "train"],

test = combatout[, whichbatch == "test"], selection = selection))

}

else if (batchCorrect == "qn")

{

dataMat <- cbind(trainExprMat, testExprMat)

dataMatNorm <- normalize.quantiles(dataMat)

whichbatch <- as.factor(c(rep("train", ncol(trainExprMat)),

rep("test", ncol(testExprMat))))

return(list(train = dataMatNorm[, whichbatch == "train"],

test = dataMatNorm[, whichbatch == "test"],

selection = selection))

}

else {

return(list(train = trainExprMat, test = testExprMat,

selection = selection))

}

}

#'This function takes a gene expression matrix and if duplicate genes are measured, summarizes them by their means.

#'@param exprMat A gene expression matrix with genes as rownames() and samples as colnames().

#'@return A gene expression matrix that does not contain duplicate genes.

#'@keywords Summarize duplicate genes by their mean.

#'@export

summarizeGenesByMean <- function(exprMat)

{

geneIds <- rownames(exprMat)

t <- table(geneIds) #How many times is each gene name duplicated.

allNumDups <- unique(t)

allNumDups <- allNumDups[-which(allNumDups == 1)]

#Create a *new* gene expression matrix with everything in the correct order....

#Starrt by just adding stuff that isn't duplicated

exprMatUnique <- exprMat[which(geneIds %in% names(t[t == 1])), ]

gnamesUnique <- geneIds[which(geneIds %in% names(t[t == 1]))]

#Add all the duplicated genes to the bottom of "exprMatUniqueHuman", summarizing as you go

for(numDups in allNumDups)

{

geneList <- names(which(t == numDups))

for(i in 1:length(geneList))

{

exprMatUnique <- rbind(exprMatUnique, colMeans(exprMat[which(geneIds == geneList[i]), ]))

gnamesUnique <- c(gnamesUnique, geneList[i])

# print(i)

}

}

if(class(exprMatUnique) == "numeric")

{

exprMatUnique <- matrix(exprMatUnique, ncol=1)

}

rownames(exprMatUnique) <- gnamesUnique

return(exprMatUnique)

}

#'This function predicts a phenotype (drug sensitivity score) when provided with microarray or bulk RNAseq gene expression data of different platforms.

#'The imputations are performed using ridge regression, training on a gene expression matrix where phenotype is already known.

#'This function integrates training and testing datasets via a user-defined procedure, and power transforming the known phenotype.

#'@param trainingExprData The training data. A matrix of expression levels. rownames() are genes, colnames() are samples (cell line names or cosmic ides, etc.). rownames() must be specified and must contain the same type of gene ids as "testExprData"

#'@param trainingPtype The known phenotype for "trainingExprData". This data must be a matrix of drugs/rows x cell lines/columns or cosmic ids/columns. This matrix can contain NA values, that is ok (they are removed in the calcPhenotype() function).

#'@param testExprData The test data where the phenotype will be estimated. It is a matrix of expression levels, rows contain genes and columns contain samples, "rownames()" must be specified and must contain the same type of gene ids as "trainingExprData".

#'@param batchCorrect How should training and test data matrices be homogenized. Choices are "eb" (default) for ComBat, "qn" for quantiles normalization or "none" for no homogenization.

#'@param powerTransformPhenotype Should the phenotype be power transformed before we fit the regression model? Default to TRUE, set to FALSE if the phenotype is already known to be highly normal.

#'@param removeLowVaryingGenes What proportion of low varying genes should be removed? 20 percent be default

#'@param minNumSamples How many training and test samples are required. Print an error if below this threshold

#'@param selection How should duplicate gene ids be handled. Default is -1 which asks the user. 1 to summarize by their or 2 to disguard all duplicates.

#'@param printOutput Set to FALSE to supress output.

#'@param pcr Indicates whether or not you'd like to use pcr for feature (gene) reduction. Options are 'TRUE' and 'FALSE'. If you indicate 'report_pc=TRUE' you need to also indicate 'pcr=TRUE'

#'@param removeLowVaringGenesFrom Determine method to remove low varying genes. Options are 'homogenizeData' and 'rawData'.

#'@param report_pc Indicates whether you want to output the training principal components. Options are 'TRUE' and 'FALSE'.

#'@param cc Indicate if you want correlation coefficients for biomarker discovery.

#'@param percent Indicate percent variability (of the training data) you'd like principal components to reflect if pcr=TRUE. Default is 80 for 80%

#'These are the correlations between a given gene of interest across all samples vs. a given drug response across samples.

#'These correlations can be ranked to obtain a ranked correlation to determine highly correlated drug-gene associations.

#'@param rsq Indicate whether or not you want to output the R^2 values for the data you train on from true and predicted values.

#'These values represent the percentage in which the optimal model accounts for the variance in the training data.

#'Options are 'TRUE' and 'FALSE'.

#'@return .txt files will be saved into your working directory. Depending on the parameter specified, the .txt file outputs of this function can include the estimated phenotype/sensitivity predictions,

#'the R^2 data, and the correlation coefficients. Principal components are stored as .RData files for each drug in your drug dataset.

#'@import sva

#'@import ridge

#'@import car

#'@import ridge

#'@import glmnet

#'@import tidyverse

#'@import utils

#'@import stats

#'@importFrom pls explvar explvar

#'@keywords predict drug sensitivity and phenotype

#'@export

calcPhenotype<-function (trainingExprData,

trainingPtype,

testExprData,

batchCorrect,

powerTransformPhenotype=TRUE,

removeLowVaryingGenes=0.2,

minNumSamples,

selection=1,

printOutput,

pcr=FALSE,

removeLowVaringGenesFrom,

report_pc=FALSE,

cc=FALSE,

percent=80,

rsq=FALSE)

{

#Initiate empty lists for each data type you'd like to collect.

#_______________________________________________________________

DrugPredictions<-list() #Collects drug predictions.

rsqs<-list() #Collects R^2 values.

cors<-list() #Collects correlation coefficient for each gene across all samples vs. each drug across all samples.

pvalues<-list() #Collects p-values for the correlation coefficients.

#vs=c()

drugs<-colnames(trainingPtype) #Store all the possible drugs in a vector.

#Check the supplied data and parameters.

#_______________________________________________________________

if (class(testExprData)[1] != "matrix")

stop("\nERROR: \"testExprData\" must be a matrix.")

if (class(trainingExprData)[1] != "matrix")

stop("\nERROR: \"trainingExprData\" must be a matrix.")

if (class(trainingPtype)[1] != "matrix")

stop("\nERROR: \"trainingPtype\" must be a matrix.")

if (report_pc)

if (pcr == FALSE)

stop("\nERROR: pcr must be TRUE if report_pc is TRUE")

if (pcr)

if (cc)

stop("\nERROR: pcr must be FALSE if cc is TRUE")

#Make sure training samples are equivalent in both matrices.

if (!any(colnames(trainingExprData) %in% rownames(trainingPtype)))

stop("\nERROR: No Cell Lines Found in Common: Sample names must be consistent in training matrices")

#Subset and order the training Expr and trainingPtype to the cell lines in common (and order them)

commonCellLines<-colnames(trainingExprData)[colnames(trainingExprData) %in% rownames(trainingPtype)]

trainingExprData <- trainingExprData[,commonCellLines]

trainingPtype <- trainingPtype[commonCellLines,]

#Check if an adequate number of training and test samples have been supplied.

#_______________________________________________________________

if ((nrow(trainingExprData) < minNumSamples) || (nrow(testExprData) < minNumSamples)) {

stop(paste("\nThere are less than", minNumSamples, "samples in your test or training set. It is strongly recommended that you use larger numbers of samples in order to (a) correct for batch effects and (b) fit a reliable model. To supress this message, change the \"minNumSamples\" parameter to this function."))

}

#Get the homogenized data.

#_______________________________________________________________

homData <- homogenizeData(testExprMat=testExprData, trainExprMat=trainingExprData, batchCorrect, selection, printOutput)

#Remove low varying genes.

#_______________________________________________________________

#Do variable selection if specified. By default, we remove 20% of least varying genes from the homogenized dataset.

#We can also remove the intersection of the lowest 20% from both training and test sets (for the gene ids remaining in the homogenized data).

#Otherwise, keep all genes.

#Check batchCorrect parameter.

if (!(removeLowVaringGenesFrom %in% c("homogenizeData", "rawData"))) {

stop("\nremoveLowVaringGenesFrom\" must be one of \"homogenizeData\", \"rawData\"")

}

keepRows <- seq(1:nrow(homData$train)) #By default we will keep all the genes.

if (removeLowVaryingGenes > 0 && removeLowVaryingGenes < 1) { #If the proportion of variability to keep is between 0 and 1.

if (removeLowVaringGenesFrom == "homogenizeData") { #If you're filtering based on homogenized data.

keepRows <- doVariableSelection(cbind(homData$test, homData$train), removeLowVaryingGenes = removeLowVaryingGenes)

numberGenesRemoved <- nrow(homData$test) - length(keepRows)

if (printOutput) message(paste("\n", numberGenesRemoved, "low variabilty genes filtered."));

}

else if (removeLowVaringGenesFrom == "rawData") { #If we are filtering based on the raw data i.e. the intersection of the things filtered from both datasets.

evaluabeGenes <- rownames(homData$test)

keepRowsTrain <- doVariableSelection(trainingExprData[evaluabeGenes,], removeLowVaryingGenes = removeLowVaryingGenes)

keepRowsTest <- doVariableSelection(testExprData[evaluabeGenes,], removeLowVaryingGenes = removeLowVaryingGenes)

keepRows <- intersect(keepRowsTrain, keepRowsTest)

numberGenesRemoved <- nrow(homData$test) - length(keepRows)

if (printOutput)

message(paste("\n", numberGenesRemoved, "low variabilty genes filtered."));

}

}

#Predict for each drug.

#_______________________________________________________________

for(a in 1:length(drugs)){ #For each drug...

#Modify trainingPtype and trainingExprData so that you only use cell lines for which you have expression and response data for.

#_______________________________________________________________

trainingPtype2<-trainingPtype[,a] #Obtain the response data for the compound of interest.

NonNAindex <- which(!is.na(trainingPtype2)) #Get the indices of the non NAs. You only want the cell lines/cosmic ids that you have drug info for.

samps<-rownames(trainingPtype)[NonNAindex] #Obtain cell lines you have expression and response data for.

if (length(samps) == 1){ #Make sure training data has more than just 1 sample. If the drug has one sample, it will be skipped.

drugs = drugs[-a]

message(paste("\n", drugs[a], "is skipped due to insufficient cell lines to fit the model."))

next

} else {

trainingPtype4<-as.numeric(trainingPtype2[NonNAindex]) #This makes sure you use all the response data for the drug without NA values.

#PowerTransform the phenotype if specified.

#_______________________________________________________________

offset = 0

if (powerTransformPhenotype){

if (min(trainingPtype4) < 0){ #All numbers must be positive for a powertransform to work, so make them positive.

offset <- -min(trainingPtype4) + 1

trainingPtype4 <- trainingPtype4 + offset

}

transForm <- powerTransform(trainingPtype4)[[6]]

trainingPtype4 <- trainingPtype4^transForm

}

#Create the ridge regression model on the training data using pcr (keeping the number of components required for 80% variance).

#_______________________________________________________________

if (pcr){

#There are many ways for pcr in R...here I will use pcr() (principal component regression).

#Use pcr to predict for testing data.

#_______________________________________________________________

train_x<-(t(homData$train)[samps,keepRows]) #samps represent the cell lines that have been filtered, keepRows represents the genes.

train_y<-trainingPtype4

test_x<-(t(homData$test)[,keepRows])

#Remove genes that you have no transcriptome data for (aka columns are filled with 0's).

#If you don't, it causes problems in linearRidge().

#This is weird, but there were 2 genes in CTRPv2 data that had 0's for one drug (I suppose this occurs depending on sample filtration).

#Make sure you remove those same genes from the test data...want to make sure the genes are the same in both train_x and test_x

x<-as.vector(colSums(train_x)) #Sum each column.

bad<-which(x == 0) #Column/genes indices that are filled with only 0's.

if(length(bad) != 0){

train_x<-train_x[,-bad]

test_x<-data.frame(test_x[,-bad])

}

#Remove genes with 0 variance. If you don't, this will also cause problems in linearRidge().

variance<-c()

for(i in 1:ncol(train_x)){

variance[i]<-var(as.vector(train_x[,i]))

}

bi<-which(variance %in% 0) #Bad index...gene has 0 variance.

if(length(bi) != 0){

train_x<-train_x[,-bi]

test_x<-data.frame(test_x[,-bi])

}

#Check to make sure you have more than 1 training sample for the drug's model.

#_______________________________________________________________

trainFrame<-try(data.frame(Resp=train_y, train_x), silent = TRUE)

if (dim(trainFrame)[1] == 1){ #Make sure you have more than 1 sample.

drugs = drugs[-a]

message(paste("\n", drugs[a], "is skipped due to insufficient cell lines to fit the model."))

next

} else {

pcr_model<-pcr(Resp~., data=trainFrame, validation='CV')

v=cumsum(explvar(pcr_model)) #A vector of all the pcs and their percent of variance.

ncomp=min(which(v > percent)) #Identify which pcs will represent 80% variance.

#vs[a]<-ncomp

if(printOutput) message("\nCalculating predicted phenotype using pcr...")

preds<-predict(pcr_model, newdata=test_x, ncomp=ncomp)

#You can compute an R^2 value for the data you train on from true and predicted values.

#The rsq value represents the percentage in which the optimal model accounts for the variance in the training data.

#_______________________________________________________________

if (rsq){

if (dim(train_x)[1] < 4){ #The code will result in an error if you have 3 samples (which is enough for the model fitting but not when you do a 70/30% split)...

message(paste("\n", drugs[a], 'is skipped for R^2 analysis'))

}else{

#trainFrame<-data.frame(Resp=train_y, train_x)

data<-(cbind(train_x, train_y)) #Rows are samples, columns are genes.

dt<-sort(sample(nrow(data), nrow(data)*.7)) #sample() randomly picks 70% of rows/samples from the dataset. It samples without replacement.

#Prepare the training data (70% of original training data)

train_x<-data[dt,]

ncol<-dim(train_x)[2]

train_y<-train_x[,ncol]

train_x<-train_x[,-ncol]

#Prepare the testing data (30% of original training data)

test_x<-data[-dt,]

ncol<-dim(test_x)[2]

test_y<-test_x[,ncol]

test_x<-test_x[,-ncol]

#Remove genes that you have no transcriptome data for aka columns are filled with 0's.

#If you don't, it causes problems in linearRidge()

#Make sure you remove these same genes from the test data...want to make sure the genes are the same in both.

x<-as.vector(colSums(train_x))

bad<-which(x == 0)

if (length(bad) != 0){

train_x<-train_x[,-bad]

test_x<-data.frame(test_x[,-bad])

}

#Remove genes with 0 variance. If you don't, this will also cause problems in linearRidge()

variance<-c()

for(i in 1:ncol(train_x)){

variance[i]<-var(as.vector(train_x[,i]))

}

bi<-which(variance %in% 0) #Bad index...gene has 0 variance.

if (length(bi) != 0){

train_x<-train_x[,-bi]

}

data<-data.frame(Resp=train_y, train_x)

pcr_model<-pcr(Resp~., data=data, validation='CV') #Set validation argument to CV.

v=cumsum(explvar(pcr_model)) #A vector of all the pcs and their percent of variance.

ncomp=min(which(v > percent)) #Identify which pcs will represent 80% variance.

pcr_pred<-predict(pcr_model, test_x, ncomp=ncomp)

if (printOutput) message("\nCalculating R^2...")

sst<-sum((test_y - mean(test_y))^2) #Compute the sum of squares total.

sse<-sum((pcr_pred - test_y)^2) #Compute the sum of squares error.

rsq_value<-1 - sse/sst #Compute the rsq value.

}

}

}

if (report_pc){

if (printOutput) message("\nObtaining principal components...")

pcs<-coef(pcr_model, comps = ncomp) #comps: numeric, which components to return.

dir.create("./calcPhenotype_Output")

path<-paste('./calcPhenotype_Output/', drugs[a], '.RData', sep="")

save(pcs, file=path)

}

} else {

#Create the ridge regression model on our training data to predict for our actual testing data without pcr.

#_______________________________________________________________

if(printOutput) message("\nFitting Ridge Regression model...");

expression<-(t(homData$train)[samps,keepRows]) #samps represent the cell lines that have been filtered, keepRows represents the genes.

test_x<-(t(homData$test)[,keepRows])

#Remove genes that you have no transcriptome data for (aka columns are filled with 0's).

#If you don't, it causes problems in linearRidge().

#This is weird, but there were 2 genes in CTRPv2 data that had 0's for one drug (I suppose this occurs depending on sample filtration).

#Make sure you remove those same genes from the test data...want to make sure the genes are the same in both train_x and test_x

x<-as.vector(colSums(expression))

bad<-which(x == 0) #Column/genes indices that are filled with only 0's.

if (length(bad) != 0){

expression<-expression[,-bad]

test_x<-data.frame(test_x[,-bad])

}

#Remove genes with 0 variance. If you don't, this will also cause problems in linearRidge().

variance<-c()

for (i in 1:ncol(expression)){

variance[i]<-var(as.vector(expression[,i]))

}

bi<-which(variance %in% 0) #Bad index...gene has 0 variance.

if (length(bi) != 0){ #If there are actually bad indices/genes that had 0 variance across all samples, remove them.

expression<-expression[,-bi]

test_x<-data.frame(test_x[,-bi])

}

#Check to make sure you have more than 1 training sample for the drug's model.

#_______________________________________________________________

trainFrame<-try(data.frame(Resp=trainingPtype4, expression), silent = TRUE)

if (dim(trainFrame)[1] == 1){ #Make sure you have more than 1 sample.

drugs = drugs[-a]

message(paste("\n", drugs[a], "is skipped due to insufficient cell lines to fit the model."))

next

} else {

if(printOutput) message("\nCalculating predicted phenotype...")

rrModel<-linearRidge(Resp ~., data=trainFrame)

preds<-predict(rrModel, newdata=data.frame(test_x))

#You can compute an R^2 value for the data you train on from true and predicted values.

#The R^2 value represents the percentage in which the optimal model accounts for the variance in the training data.

#_______________________________________________________________

if(rsq){

if (dim(expression)[1] < 4){ #The code will result in an error if you have 3 samples...this makes sure you have more than 3 samples.

#It results in an error because there is a 70/30% split of training data.

message(paste("\n", drugs[a], 'is skipped for R^2 analysis'))

} else {

expression<-(cbind(expression, trainingPtype4))

dt<-sort(sample(nrow(expression), nrow(expression)*.7)) #sample() randomly picks 70% of rows/samples from the dataset. It samples without replacement.

#Prepare the training data (70% of original training data)

train_x<-expression[dt,]

ncol<-dim(train_x)[2]

train_y<-train_x[,ncol]

train_x<-train_x[,-ncol]

#Prepare the testing data (30% of original training data)

test_x<-expression[-dt,]

ncol<-dim(test_x)[2]

test_y<-test_x[,ncol]

test_x<-test_x[,-ncol]

#Remove genes that you have no transcriptome data for (aka columns are filled with 0's).

#If you don't, it causes problems in linearRidge().

#Make sure you remove those same genes from the test data...want to make sure the genes are the same in both train_x and test_x

x<-as.vector(colSums(train_x))

bad<-which(x == 0) #Column/genes indices that are filled with only 0's.

if (length(bad) != 0){

train_x<-train_x[,-bad]

test_x<-data.frame(test_x[,-bad])

}

#Remove genes with 0 variance. If you don't, this will also cause problems in linearRidge().

variance<-c()

for (i in 1:ncol(train_x)){

variance[i]<-var(as.vector(train_x[,i]))

}

bi<-which(variance %in% 0) #Bad index...gene has 0 variance.

if (length(bi) != 0){ #If there are actually bad indices/genes that had 0 variance across all samples, remove them.

train_x<-train_x[,-bi]

test_x<-data.frame(test_x[,-bi])

}

trainFrame<-data.frame(Resp=train_y, train_x)

rrModel<-linearRidge(Resp ~., data=trainFrame)

testFrame<-data.frame(test_x)

pred<-predict(rrModel, newdata=testFrame)

if(printOutput) message("\nCalculating R^2...")

sst<-sum((test_y - mean(test_y))^2) #Compute the sum of squares total.

sse<-sum((pred - test_y)^2) #Compute the sum of squares error.

rsq_value<-1 - sse/sst #Compute the rsq value.

}

}

}

}

#If the response variable was transformed (aka powerTransformPhenotype=TRUE), untransform it here.

#_______________________________________________________________

if(powerTransformPhenotype) {

preds <- preds^(1/transForm)

preds <- preds - offset

}

#Find correlation between imputed response and expression of a gene.

#Each gene is corrected differently; therefore, it may not be ideal to determine weight or percentage or weight in which each gene contributes to the prediction.

#_______________________________________________________________

if(cc){ #You can only collect correlations if pcr=FALSE!

if(pcr){

stop('ERROR: pcr must equal FALSE in order to compute correlations') #It doesn't make sense to compute correlations when the features have changed from genes to pcs.

}

if(printOutput) message("\nCalculating correlation coefficients...") #This is only relevant if you aren't using pcr.

cors_vec<-c() #This vector will store the correlation coefficients.

cors_vec2<-c() #This vector will store the p values.

matrix<-homData$test[keepRows,] #Matrix of genes x cell lines/cosmic ids.

for(d in 1:nrow(matrix)){ #For each gene...

cors_vec[d]<-cor.test(as.vector(matrix[d,]), as.vector(preds))$estimate #Compute correlation coefficient for expression of a given gene across patients vs.imputed values for a given drug across patients.

cors_vec2[d]<-cor.test(as.vector(matrix[d,]), as.vector(preds))$p.value

}

#indices<-order(cor, decreasing=TRUE)

#ordered_cor<-cor[indices] #Order the correlation coefficients from big to small.

#genes<-rownames(matrix)

#ordered_genes<-genes[indices]

#names(ordered_cor)<-ordered_genes

}

if(printOutput) message(paste("\nDone making prediction for drug", a, "of", ncol(trainingPtype)))

#Store the data in your lists.

#_______________________________________________________________

DrugPredictions[[a]]<-preds

if(rsq){

rsqs[[a]]<-rsq_value

}

if(cc){

cors[[a]]<-cors_vec

pvalues[[a]]<-cors_vec2

}

}

}

#Time to save the data!

#_______________________________________________________________

#Save drug prediction data to your home directory as a .txt file.

names(DrugPredictions)<-drugs

DrugPredictions_mat<-do.call(cbind, DrugPredictions)

colnames(DrugPredictions_mat)<-drugs

rownames(DrugPredictions_mat)<-colnames(testExprData)

dir.create("./calcPhenotype_Output")

write.csv(DrugPredictions_mat, file="./calcPhenotype_Output/DrugPredictions.csv", row.names = TRUE, col.names = TRUE)

#If rsq=TRUE, save R^2 data.

if(rsq){

names(rsqs)<-drugs

rsqs_mat<-do.call(cbind, rsqs)

dir.create("./calcPhenotype_Output")

write.table(rsqs_mat, file="./calcPhenotype_Output/R^2.txt")

}

#If CC=TRUE, save correlation coefficient data.

if(cc){

names(cors)<-drugs

cor_mat<-do.call(cbind, cors)

rownames(cor_mat)<-rownames(homData$train[keepRows,NonNAindex])

colnames(cor_mat)<-drugs

dir.create("./calcPhenotype_Output")

write.table(cor_mat, file="./calcPhenotype_Output/cors.txt")

names(pvalues)<-drugs

p_mat<-do.call(cbind, pvalues)

rownames(p_mat)<-rownames(homData$train[keepRows, NonNAindex])

colnames(p_mat)<-drugs

dir.create("./calcPhenotype_Output")

write.table(p_mat, file="./calcPhenotype_Output/pvalues.txt")

}

#print(vs)

}
